# Integrated Metabolomics and Molecular Docking Reveal Spatial and Developmental Variations in Flavor and Bioactive Constituents of *Lonicera japonica* Flos

**DOI:** 10.3390/foods15101619

**Published:** 2026-05-07

**Authors:** Weiran Feng, Zongshuo Li, Xi Dai, Yanxia Shu, Chao Yu, Yanwen Wu, Weidong Li

**Affiliations:** 1 School of Chinese Materia Medica, Beijing University of Chinese Medicine, Beijing 102488, China; fwrqml0526@163.com (W.F.); dx785074105@163.com (X.D.); shuyanxia66@163.com (Y.S.); yuchao202111@163.com (C.Y.); wuyanwen2014@163.com (Y.W.); 2 School of Life Sciences, Beijing University of Chinese Medicine, Beijing 102488, China; lizongshuo2009@126.com

**Keywords:** *Lonicera japonica*, flavor, floral organs, composition, network pharmacology, molecular docking, functional foods

## Abstract

*Lonicera japonica* Thunb. possesses significant potential for applications in beverages and functional foods. Nevertheless, most studies focus on the overall quality of flower buds, with limited comparisons across different developmental stages and distinct floral parts. This study systematically investigated changes in volatile flavor compounds and non-volatile bioactive constituents in whole flowers, calyxes, corollas, and reproductive organs before and after flowering. An integrated approach combining metabolomics profiling, entropy weight analysis, correlation network analysis, and molecular docking was employed to evaluate their potential in functional food development. The level of 3-decyn-2-ol increased markedly after flowering, with an approximately 14-fold increase in reproductive organs. Phenolic acids were highly enriched in the calyx, reaching up to 12-fold higher than in other parts. Flavonoids predominated in the corolla at levels 1.5–3-fold higher than in other tissues. Following flowering, the overall levels of phenolic acids and flavonoids decreased, while total sugars, reducing sugars, and polysaccharides increased by approximately 59%, 98%, and 35%, respectively. These results suggest that open flowers may exhibit enhanced potential for functional food applications. Entropy weight analysis indicated that the calyx contributed most to the integrated evaluation of flavor and functional attributes. Correlation network analysis identified chlorogenic acid, neochlorogenic acid, rutin, luteoloside, loganic acid, and secologanoside as key constituents, which showed potential interactions with inflammation- and immunity-related targets in molecular docking. These findings suggest that although medicinal use decreased after flowering, the edible value of *L. japonica* may increase, providing a basis for its rational utilization in functional food development.

## 1. Introduction

The dry buds or flowers of *Lonicera japonica* Thunb (LJT) at their initial bloom are called *Lonicerae japonicae* flos (LJF) [1]. LJF is a highly regarded botanical with dual applications in both food and traditional medicine [2]. In recent years, LJF has also attracted increasing attention in the food industry [3]. It is commonly consumed as an herbal tea, incorporated into porridge, or utilized in various traditional preparations, and is associated with health-promoting effects [4,5]. For example, it has been incorporated into polyphenol-rich smoothies, which have shown enhanced antioxidant activity; however, achieving sensory acceptance remains a challenge [6]. Fermented beverages produced with probiotics, such as *Lactobacillus acidophilus*, have demonstrated improved bioavailability of phenolic compounds and enhanced functional properties [7]. Additionally, LJF is widely used in tea-based products, including kombucha, where fermentation may alter its bioactive composition and sensory properties [8]. Commercial products such as herbal beverages and honeysuckle drinks are also readily available, reflecting the growing market demand and underscoring the necessity for standardized processing and quality control [9,10,11].

LJT is extensively cultivated in East Asia, where large-scale production systems are well-established [12,13]. However, as a specialty medicinal and edible plant, comprehensive and standardized global production data remain limited. LJF is rich in phenolic acids, flavonoids, and iridoids, which contribute to its diverse biological activities, including anti-inflammatory, antioxidant, and antimicrobial effects [14,15]. These constituents are also regarded as significant contributors to its potential value as a functional food ingredient. However, under conventional harvesting practices, only flower buds are collected for medicinal use, whereas fully opened flowers are rarely utilized, leading to the underutilization of potentially valuable plant resources [16].

The developmental stage of a plant is a critical factor influencing metabolite accumulation and quality formation. During floral development, dynamic metabolic changes occur, resulting in significant shifts in both volatile and bioactive constituents [17]. Volatile compounds primarily contribute to flavor quality, while non-volatile secondary metabolites, such as phenolic acids and flavonoids, largely account for biological activities [18,19]. Previous studies on LJF have mainly focused on identifying volatile compounds in whole flowers or quantitatively determining major bioactive constituents at the bud stage. Liu et al. [20] employed GC-IMS to systematically characterize volatile and non-volatile components in different grades of LJF, identifying 12 differential volatile metabolites, including 2-phenylethanol, methional, and benzothiazole. Zhang et al. [21] established an HPLC fingerprint combined with multi-component quantification and chemometric analysis to evaluate 39 batches of LJF from three major producing regions (Shandong, Henan, and Hebei). They constructed a fingerprint containing 24 common peaks and quantitatively determined 19 constituents. Multivariate statistical analysis effectively discriminated between samples from different origins, with chlorogenic acid, loganic acid, and 3,5-dicaffeoylquinic acid identified as key differential markers. Despite these advances, systematic comparisons of volatile and non-volatile metabolites across different developmental stages remain limited. More importantly, the spatial distribution patterns of these metabolites among distinct floral organs (calyx, corolla, and reproductive structures) have not yet been fully elucidated.

Functional differentiation among floral parts is often reflected in distinct metabolic profiles. The calyx primarily acts as a protective structure [22], the corolla enhances sensory quality [23], and the reproductive organs (stamens and pistils) are closely associated with floral maturation [24]. However, existing studies have predominantly focused on comparisons among different plant organs of LJF, such as flowers, leaves, and stems. Li et al. [25] compared the chemical compositions and anti-inflammatory activities of flower buds, leaves, and stems, reporting a generally similar phytochemical profile across these tissues. Notably, leaves contained higher levels of hydroxycinnamic acids and flavonoids than flower buds and exhibited stronger anti-inflammatory activity, suggesting that leaves and stems could serve as complementary resources. Zhang et al. [26] employed UPLC-Q-Exactive-Orbitrap-MS and UPLC-QQ-MS/MS to systematically compare the chemical constituents of four medicinal parts (flowers, stems, leaves, and fruits) of LJF. Significant compositional differences were observed, with ten major differential compounds identified, including chlorogenic acid, secologanic acid, isochlorogenic acid A, loganin, cynaroside, loganic acid, secoxyloganin, sweroside, luteolin, and rhoifolin. Nevertheless, systematic investigations that further subdivide the flower into distinct anatomical parts (calyx, corolla, and reproductive organs) remain limited. More importantly, it is unclear how primary metabolites (e.g., sugars), volatile compounds, and secondary metabolites coordinately influence flavor characteristics and potential functional properties of LJF in a food context. This knowledge gap restricts a comprehensive understanding of quality formation and hinders the efficient utilization of different floral parts. Therefore, we hypothesize that distinct floral organs undergo differential metabolic transformations from the bud stage to the full-bloom stage, and different organs may exhibit specific metabolic profiles that contribute variably to flavor characteristics and the accumulation of bioactive constituents relevant to food applications.

This study aims to systematically evaluate the dynamic distribution of flavor and bioactive constituents in LJF across two developmental stages and three distinct floral organs. By integrating headspace solid-phase microextraction coupled with gas chromatography–mass spectrometry (HS-SPME-GC–MS) and targeted metabolomics with the entropy weight method (EWM), we assessed the spatiotemporal variations and organ-specific contributions. Furthermore, correlation networks and molecular docking were employed to explore the potential mechanisms of core metabolic markers. These findings provide a scientific foundation for the differentiated utilization of LJF, offering insights into ingredient selection for functional foods that balance nutritional value and sensory quality.

## 2. Materials and Methods

### 2.1. Materials

Juhua No. 1 LJT was collected in June 2021 from a standardized cultivation base located in Jijiazhai Village, Julu County, Hebei Province, China. Samples were harvested at two developmental stages: the bud stage and the full-bloom stage. The plant materials were identified as the dried buds and flowers of LJF. Based on structural and morphological characteristics, each flower was manually separated into three distinct anatomical parts: the corolla, the reproductive organs (stamens and pistils), and the calyx. All samples were stored at −4 °C prior to further analysis.

### 2.2. HS-GC-MS Analysis

Whole flowers and their separated floral parts, collected at both the bud and full-bloom stages, were rapidly frozen in liquid nitrogen and ground into a fine, homogeneous powder using a laboratory ball mill (Ningbo Scientz Biotechnology Co., Ltd., Ningbo, China) to ensure sample uniformity and representativeness. For each sample, three independent portions (50 mg each) of the homogenized powder were accurately weighed into 10 mL headspace vials, which were then immediately sealed with caps and subjected to instrumental analysis. All experiments were conducted with three independent biological replicates (n = 3). Headspace incubation took place at 100 °C for 25 min. The quantitative loop temperature was set to 120 °C, while the transfer line temperature was maintained at 150 °C. The GC cycle time was 60 min, and the injection volume was 1 mL.

Chromatographic separation was performed using an Agilent 19091S-433UI HP-5ms Ultra Inert capillary column (30 m × 250 μm × 0.25 μm) (Agilent Technologies, Santa Clara, CA, USA). High-purity helium (>99.999%) was employed as the carrier gas under constant-flow conditions at a rate of 1.0 mL·min^−1^. The injector temperature was set to 230 °C with a split ratio of 5:1. The initial oven temperature was 40 °C and held for 5 min, followed by a ramping increase to 100 °C at a rate of 5 °C·min^−1^, and then further increased to 280 °C at a rate of 4 °C·min^−1^, where it was maintained for 8 min. Mass spectrometric detection was performed in positive electron ionization (EI) mode at 70 eV. The ion source temperature was set to 230 °C, and the mass scanning range was *m*/*z* 30–550. The solvent delay time was established at 0 min.

### 2.3. UPLC-Q-Extractive Orbitrap-MS Analysis

The different parts of LJF were rapidly frozen in liquid nitrogen and then ground into a homogeneous powder. For each sample, three independent biological replicates (n = 3) were prepared. Samples of 100 mg were accurately measured and placed into 2 mL Eppendorf tubes, followed by the addition of 1.0 mL of 70% methanol. The tubes were then sealed, subjected to ultrasonication for 45 min, and centrifuged for 10 min at 10,000 r/min. The supernatant was then transferred into a clean vial for further use.

Qualitative and quantitative analyses were performed using a UHPLC DIONEX Ultimate 3000 instrument coupled with a Q-Extractive mass spectrometer (Thermo Fisher Scientific, Waltham, MA, USA), equipped with an ACQUITY UPLC HSS T3 column (2.1 mm × 150 mm, 1.8 μm; Waters, Milford, MA, USA). The chromatographic conditions were as follows: flow rate, 0.3 mL/min; column temperature, 25 °C; injection volume, 2 μL; mobile phase A, 0.1% (*v*/*v*) formic acid in ultrapure water; mobile phase B, 0.1% (*v*/*v*) formic acid in acetonitrile. The gradient program was set as follows: 0–4 min, 98% A; 4–5 min, 98–90% A; 5–14 min, 90–82% A; 14–18 min, 82–75% A; 18–19 min, 75–56% A; 19–22 min, 56–52% A; 22–27 min, 52–28% A; 27–30 min, 28–5% A; 30–36 min, 5% A. The MS conditions were as follows: HR-ESI ion source; capillary voltage 3500 V in positive ion mode and 2800 V in negative ion mode; capillary temperature 320 °C; source temperature 350 °C; scanning range *m*/*z* 50–1500; resolution: 70,000; S-Lens RF Level: 50; sheath gas flow rate: 35 arb; auxiliary gas flow rate: 10 arb.

Metabolites were characterized based on HRAM and isotopic patterns via Thermo Xcalibur, with a mass tolerance of <5 ppm. The structural assignment was derived from MS/MS fragment ion analysis and verified through database searches (METLIN, MassBank, and HMDB) alongside comparison with literature data. This integrative approach ensured the systematic identification of chemical constituents in various organs of LJF.

### 2.4. Quantitative Determination of Phenolic Acids, Flavonoids, and Iridoids

Approximately 100 mg of each powdered sample from three independent biological replicates (n = 3) was accurately weighed into a 10 mL centrifuge tube and extracted with 5.0 mL of 70% methanol. The mixture was ultrasonicated for 45 min (power: 250 W; frequency: 40 kHz) and then allowed to stand at room temperature for 1 h. After centrifugation at 10,000 r·min^−1^ for 10 min, the supernatant was collected for subsequent analysis. Chromatographic separation was performed using a Diamonsil Plus C18 column (250 mm × 4.6 mm, 5 μm) (Dikma Technologies Inc., Beijing, China). The flow rate was maintained at 1.0 mL·min^−1^, the column temperature was set at 25 °C, and the injection volume was 10 μL. The mobile phase consisted of acetonitrile (A) and 0.2% phosphoric acid aqueous solution (B). Gradient elution was conducted as follows: 0–15 min, 5–10% A; 15–45 min, 10–11% A; 45–60 min, 11–16% A; 60–80 min, 16–17% A; 80–95 min, 17–30% A; and 95–100 min, 30–90% A. The method was validated in terms of linearity, precision, and repeatability. Detailed validation results, including calibration curves, correlation coefficients, LOD, and LOQ values, are provided in Supplementary Materials (text) and Table S1.

### 2.5. Determination of Sugar Components

Total sugar content was determined using the phenol–sulfuric acid method, while reducing sugars were measured by the 3,5-dinitrosalicylic acid (DNS) colorimetric method in accordance with GB/T 5009.7-2016 [27]. The content of polysaccharides was calculated by subtracting the reducing sugar content from the total sugar content.

### 2.6. Quantitative Contribution Analysis Based on EWM

To systematically evaluate the relative contributions of the calyx, corolla, and reproductive organs to the flavor quality and functional value of LJT, a comprehensive evaluation model based on the EWM was established. By combining objective weight determination based on information entropy with linear weighting, the comprehensive score of each floral organ was calculated.

The analysis followed a structured workflow: (1) normalization of indicators; (2) EWM-based determination of indicator weights; and (3) calculation of comprehensive scores via weighted linear summation. All calculations were performed using SPSS and Microsoft Excel. Detailed formulas and computational procedures are provided in the Supplementary Materials. This evaluation method enables precise quantification of the contribution of each floral organ to the overall quality, thereby providing a scientific basis for the differentiated utilization of *Lonicera japonica* resources.

### 2.7. Network Pharmacology and Molecular Docking Analysis

Potential targets of differential metabolites in LJF were initially predicted using platforms such as SwissTargetPrediction. The identified targets were subsequently imported into the STRING database to construct a protein–protein interaction (PPI) network, which was then visualized using Cytoscape version 3.9.1. Gene Ontology (GO) functional annotation and Kyoto Encyclopedia of Genes and Genomes (KEGG) pathway enrichment analyses were performed to explore the biological roles of the predicted targets. For molecular docking, the three-dimensional structures of the active compounds and the key protein targets were obtained from the PubChem and Protein Data Bank (PDB) databases, respectively. Docking simulations were conducted using AutoDock 1.5.7 Vina, and the resulting docking conformations were visualized with PyMOL 3.0.3 to assess potential binding interactions.

### 2.8. Statistical Analysis

SPSS version 19.0 (SPSS Inc., Chicago, IL, USA) was used for statistical analysis. Data are presented as mean ± standard deviation (SD). Two-way analysis of variance (ANOVA) was performed to evaluate the effects of developmental stage (Stage), floral organ (Organ), and their interaction (Stage × Organ). When significant differences were detected, post hoc multiple comparisons were conducted to assess differences among groups. A *p* < 0.05 was considered statistically significant. Origin 2026 (OriginLab, Northampton, MA, USA) was used for data visualization.

**Figure 1:**
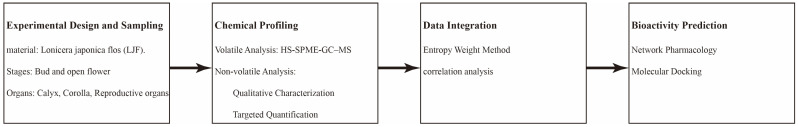
Schematic overview of the integrated analytical workflow for LJF. The diagram illustrates the comprehensive experimental process, encompassing sample collection and preparation, integrated profiling of volatile and non-volatile metabolites, systematic quality assessment via the EWM, and potential function prediction through molecular docking.

To provide a clear overview of the integration of the sampling, analytical, and evaluative methods, the comprehensive experimental scheme is summarized in Figure 1.

## 3. Results

### 3.1. Changes in Volatile Flavor Compounds Before and After Flowering

**Figure 2:**
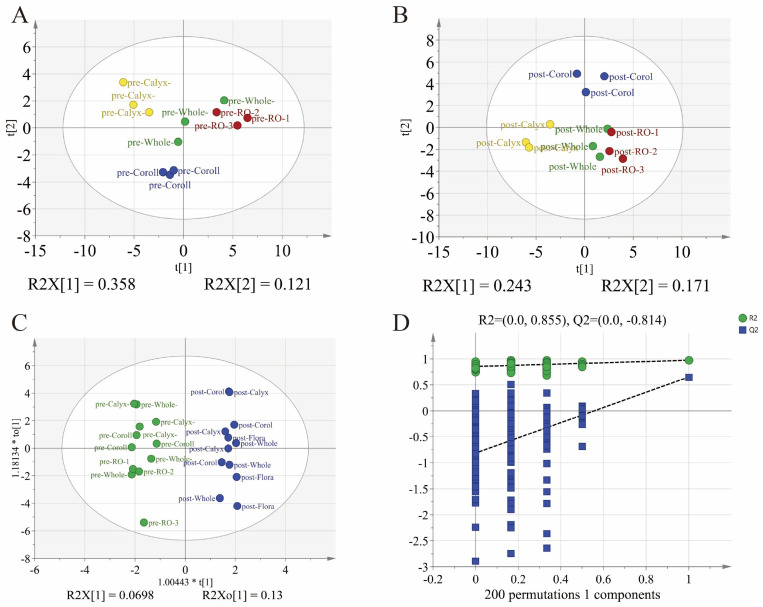
Comparative analysis of volatile compounds. (**A**) PLS-DA score plot showing differences among floral organs before flowering; (**B**) PLS-DA score plot showing differences among floral organs after flowering; (**C**) OPLS-DA score plot illustrating differences in volatile metabolites between pre- and post-flowering stages; (**D**) Permutation test for validation of the OPLS-DA model.

A total of 41 volatile compounds were identified across the various floral organs of LJF, including 13 aldehydes, 13 alcohols, 5 esters, 4 acids, and 6 other compounds. The composition and relative abundances of volatile metabolites in each organ, both before and after flowering, are summarized in Table S2. To thoroughly assess the differences in volatile compounds among floral organs and developmental stages, partial least squares discriminant analysis (PLS-DA) and orthogonal PLS-DA (OPLS-DA) were applied to the GC–MS data. As illustrated in Figure 2A,B, samples from different organs exhibited clear separation in the score plots, both before and after flowering. Notable differences in volatile metabolites were evident among the organs prior to flowering. Following flowering, the organ-specific metabolic activity remained pronounced, with the reproductive organs displaying a profile closely resembling that of whole flowers. This suggests that the reproductive organs contribute significantly to the overall floral aroma at the full-bloom stage. The OPLS-DA model focusing on whole flowers before flowering (Figure 2C) confirmed that developmental stage is a major factor influencing the variation in volatile metabolites. Additionally, model validation through permutation testing (n = 200) further confirmed its reliability (Figure 2D), indicating that the observed differences in volatile profiles before and after flowering are robust and statistically significant.

**Figure 3:**
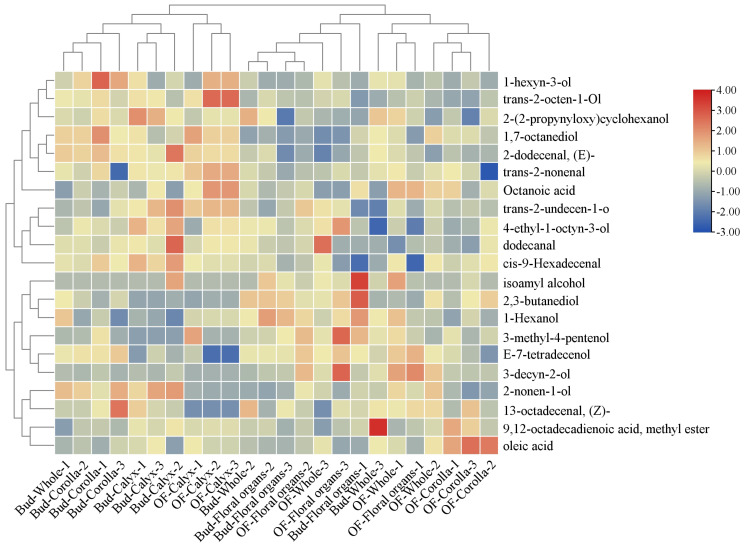
Hierarchical clustering heatmap of differential volatile compounds.

**Figure 4:**
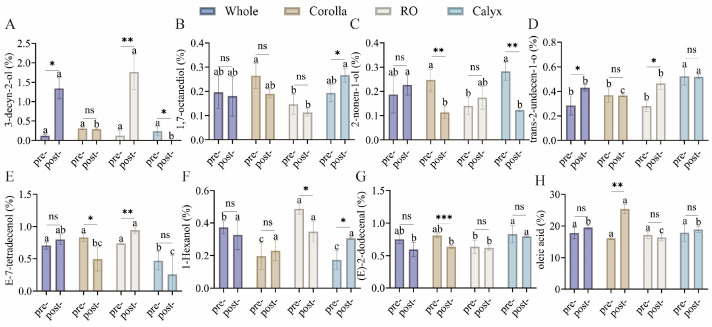
Differential volatile compounds in three floral organs at two developmental stages. (**A**) 3-decyn-2-ol; (**B**) 1,7-octanediol; (**C**) 2-nonen-1-ol; (**D**) trans-2-undecen-1-ol; (**E**) E-7-tetradecenol; (**F**) 1-hexanol; (**G**) (E)-2-dodecenal; (**H**) oleic acid. Data are presented as mean ± SD (n = 3). Different lowercase letters indicate significant differences among groups within the same developmental stage (*p* < 0.05). Asterisks indicate significant differences between developmental stages within the same group (* *p* < 0.05, ** *p* < 0.01, *** *p* < 0.001, “ns” indicates no significant difference). Statistical analysis was performed using two-way ANOVA followed by post hoc multiple comparisons.

To visualize the accumulation patterns of differential volatile metabolites across various stages and organs, hierarchical clustering was conducted on metabolites with VIP > 1, fold change > 1.2, and *p* < 0.05 (Figure 3). Two major clusters emerged corresponding to the pre- flowering and post-flowering stages. The calyx exhibited minimal change, whereas the whole flower and reproductive organs clustered closely after flowering, indicating an increased contribution of stamens and pistils to the overall floral aroma. Eight representative volatiles were highlighted for their contributions and biological significance (Figure 4). Oleic acid increased 1.5-fold in the corolla after flowering. 3-Decyn-2-ol was nearly undetectable at the bud stage but increased approximately 14-fold in the reproductive organs after flowering. Trans-2-undecen-1-ol and (E)-2-dodecenal maintained high levels in the calyx, contributing floral, rose-like, waxy, fatty, and citrus aromas [28]. Other volatiles, including 1-hexanol, 2-nonen-1-ol, and 1,7-octanediol, were broadly distributed across organs, suggesting their role as foundational aroma components. These differences were further supported by two-way ANOVA, which revealed significant effects of developmental stage, floral organ, and their interaction (Table S3).

### 3.2. Changes in Non-Volatile Functional Compounds Before and After Flowering

A total of 151 compounds were identified in LJF, including 46 phenolic acids, 28 flavonoids, 40 iridoids, 1 triterpenoid, 10 amino acids, 5 nucleosides, 4 fatty acids, and 17 additional compounds. The retention times, relative molecular masses, molecular formulas, and fragment ions from secondary mass spectrometry of these 151 compounds are presented in Table S4. The chemical constituents across the different parts of LJF were found to be similar, containing phenolic acids, flavonoids, and iridoids. This similarity suggests that the synthesis of these secondary metabolites occurs throughout all parts of LJF. Isomers such as neochlorogenic acid and cryptochlorogenic acid were distinguished by their characteristic MS/MS fragment ions (e.g., *m*/*z* 173.0454 vs. 191.0560). Detailed fragmentation pathways and MS/MS spectra for all major compounds are provided in Supplementary Materials (text) and Figures S1–S3.

**Figure 5:**
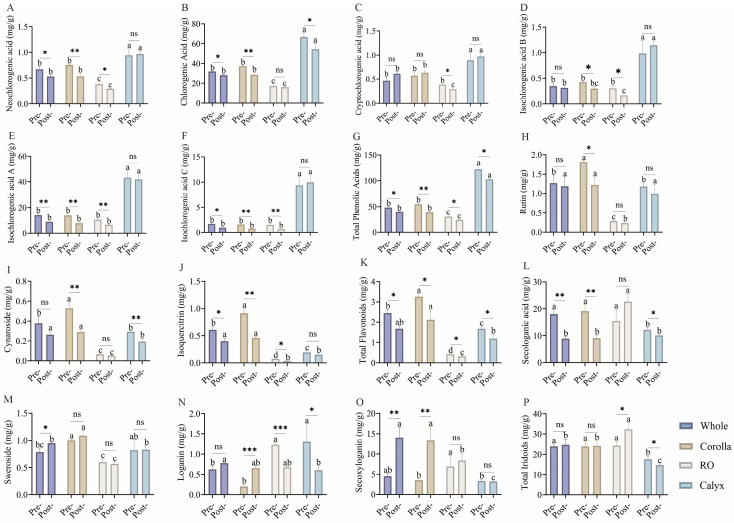
Changes in phenolic acids, flavonoids, and iridoids in different floral organs before and after flowering. (**A**) Neochlorogenic acid; (**B**) Chlorogenic acid; (**C**) Cryptochlorogenic acid; (**D**) Isochlorogenic acid B; (**E**) Isochlorogenic acid A; (**F**) Isochlorogenic acid C; (**G**) Total phenolic acids. (**H**) Rutin; (**I**) Cynaroside; (**J**) Isoquercitrin; (**K**) Total flavonoids. (**L**) Secologanic acid; (**M**) Sweroside; (**N**) Loganin; (**O**) Secoxyloganin; (**P**) Total iridoids. Data are presented as mean ± SD (n = 3). Different lowercase letters indicate significant differences among groups within the same developmental stage (*p* < 0.05). Asterisks indicate significant differences between developmental stages within the same group (* *p* < 0.05, ** *p* < 0.01, *** *p* < 0.001, “ns” indicates no significant difference). Statistical analysis was performed using two-way ANOVA followed by post hoc multiple comparisons.

Phenolic acids, flavonoids, and iridoids are the principal bioactive components responsible for the medicinal properties of LJF [29]. Targeted metabolomics was utilized to quantitatively analyze 13 individual compounds at two developmental stages (bud stage and full-bloom stage) and across four floral parts (whole flower, corolla, reproductive organs, and calyx) (Figure 5). Distinct organ-specific distribution patterns were observed among these three classes of bioactive compounds. Phenolic acids were highly enriched in the calyx. For instance, the chlorogenic acid content in the calyx (63.90–72.06 mg/g DW at the bud stage) was 3.5–4.9 times higher than that in the reproductive organs, while isochlorogenic acids A and C also reached peak concentrations in the calyx (39.31–50.32 and 7.34–10.47 mg/g DW, respectively). Iridoids, characteristic of *Lonicera* species, demonstrated organ-specific accumulation in the reproductive organs, with an increasing trend following flowering. Loganic acid, a representative iridoid, has been reported to possess anti-inflammatory and antimicrobial activities, suggesting that the post-flowering enrichment of iridoids in the reproductive organs may enhance bacterial defense [30].

After flowering, the overall contents of phenolic acids and flavonoids decreased. The total phenolics in whole flowers declined from 45.58–50.82 to 38.12–42.70 mg/g DW, calyx total phenolics decreased from 114.07–127.84 to 100.96–104.69 mg/g DW, and corolla total flavonoids dropped from 3.07–3.38 to 1.81–2.70 mg/g DW. The observed variations were confirmed by two-way ANOVA, showing significant main and interaction effects (Table S5). These findings support the traditional rationale for harvesting flower buds for medicinal purposes.

**Figure 6:**
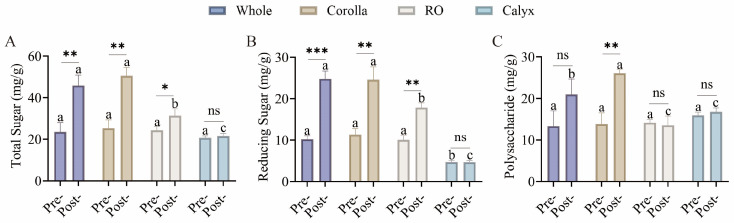
Changes in sugar contents in different floral organs before and after flowering. (**A**) Total sugars; (**B**) Reducing sugars; (**C**) Polysaccharides. Data are presented as mean ± SD (n = 3). Different lowercase letters indicate significant differences among groups within the same developmental stage (*p* < 0.05). Asterisks indicate significant differences between developmental stages within the same group (* *p* < 0.05, ** *p* < 0.01, *** *p* < 0.001, “ns” indicates no significant difference). Statistical analysis was performed using two-way ANOVA followed by post hoc multiple comparisons.

Sugars, as primary metabolites, provide essential energy and functional potential for food applications. The contents of total sugars, reducing sugars, and polysaccharides were quantified to assess the nutritional potential of different organs before and after flowering (Figure 6). All sugar fractions exhibited significant increases after flowering. Total sugars in whole flowers rose from 20.35–21.52 to 39.94–47.77 mg/g DW (1.9–2.2-fold), reducing sugars increased from 9.43–10.09 to 23.23–24.26 mg/g DW (~2.4-fold), and polysaccharides increased from 10.92–11.44 to 16.70–23.51 mg/g DW (1.5–2.1-fold). Two-way ANOVA further demonstrated significant contributions of both factors and their interaction to sugar variation (Table S6). This increase may indicate a greater potential for applications in the development of nutritional and functional foods.

### 3.3. Contribution Rates and Comprehensive Scores of Floral Organs Calculated by the EWM

**Figure 7:**
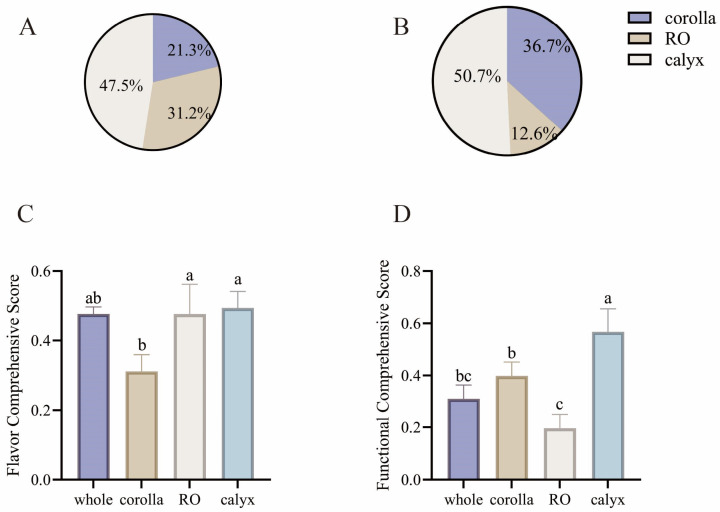
Contribution rates and comprehensive scores calculated using the entropy weight method and linear weighted method. (**A**) Flavor contribution rate; (**B**) Functional contribution rate; (**C**) Comprehensive flavor score; (**D**) Comprehensive functional score. Data are presented as mean ± SD (n = 3). Different lowercase letters above the bars indicate significant differences among groups (*p* < 0.05). Statistical analysis was performed using one-way ANOVA followed by multiple comparisons.

To quantitatively evaluate the relative importance of different floral organs in LJF concerning flavor quality and functional value, the EWM was applied to the measured volatile and non-volatile metabolites. Weighted analyses were utilized to calculate organ-specific contributions to flavor, function, and overall scores (Figure 7). The calyx exhibited the highest contribution to overall floral aroma, accounting for 47.5%, followed by the reproductive organs at 31.2%, while the corolla contributed 21.3%. The dominant flavor contribution of the calyx is likely attributable to the sustained high accumulation of aldehydes such as trans-2-undecen-1-ol and (E)-2-dodecenal, whereas organ-specific metabolites in the reproductive organs, including 3-decyn-2-ol, contributed secondary aroma characteristics. In terms of functional value, the calyx also dominated with a contribution of 50.7%, followed by the corolla at 36.7%, and the reproductive organs at 12.6%. Phenolic acids enriched in the calyx served as the primary source of functional contribution, while flavonoids accumulated in the corolla, including rutin and cynaroside, provided secondary functional value. These findings were consistent with the calculated comprehensive scores, demonstrating that the calyx is the core contributor to both flavor and functional quality, the reproductive organs mainly contribute to aroma, and the corolla primarily supports functional properties. This quantitative evaluation provides a scientific basis for the differentiated utilization of LJF organs. Specifically, the calyx is suitable for dual-purpose development targeting both flavor and functional value, the reproductive organs can be selectively employed for flavor-focused products, and the corolla is optimal for the extraction of bioactive functional compounds.

### 3.4. Correlation Between Functional Metabolites and Potential Bioactivity Indicators

**Figure 8:**
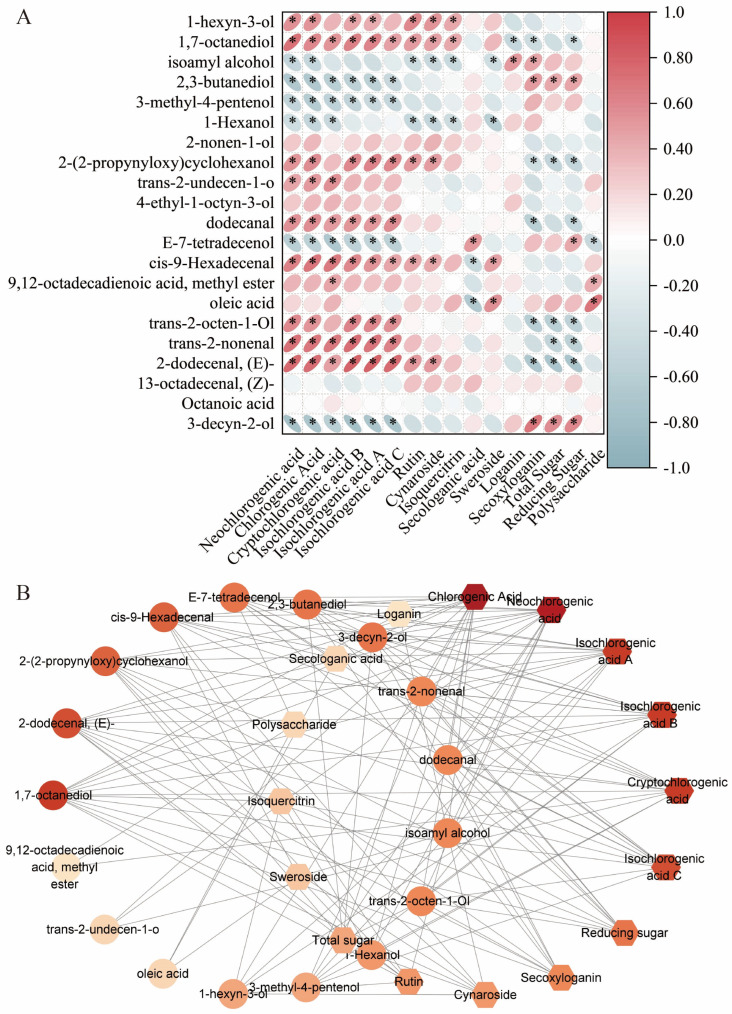
Correlation analysis of polysaccharides, total acids, total flavonoids, iridoids, and volatile compounds. (**A**) Correlation heatmap; (**B**) Correlation network analysis. The color intensity indicates the degree value, with darker colors representing higher degree values and greater importance of the components. “*” indicate significant correlations (*p* < 0.05).

To investigate the intrinsic relationships between flavor compounds and functional metabolites in LJF, we conducted Spearman correlation analyses on volatile metabolites, phenolic acids, flavonoids, iridoids, and sugars to identify key bioactive markers. A metabolite association network was then constructed based on the resulting correlation matrix (Figure 8). Notably, chlorogenic acid and neochlorogenic acid exhibited strong correlations with aldehydes such as (E)-2-dodecenal, trans-2-nonenal, and 2-dodecena (*p* < 0.01). These compounds were co-enriched in the calyx, supporting its role in providing a protective chemical barrier, where antimicrobial phenolic acids and volatile aldehyde defense compounds accumulate synergistically to form a protective barrier. Flavonoids such as rutin and cynaroside showed associations with certain alcohols and oleic acid. Secoxyloganin displayed strong correlations with total sugar, reducing sugar, and polysaccharides, positioning it as a central node linking primary metabolites and iridoid secondary metabolites. Secologanic acid served as a connector between iridoids and multiple volatile compounds. Based on network centrality and degree values, six key candidate markers, including chlorogenic acid, cynaroside, neochlorogenic acid, rutin, secologanic acid, and secoxyloganin, were selected for subsequent molecular docking analyses to explore their potential molecular mechanisms underlying the pharmacological activity of LJF.

### 3.5. Network Pharmacology and Molecular Docking Results

**Figure 9:**
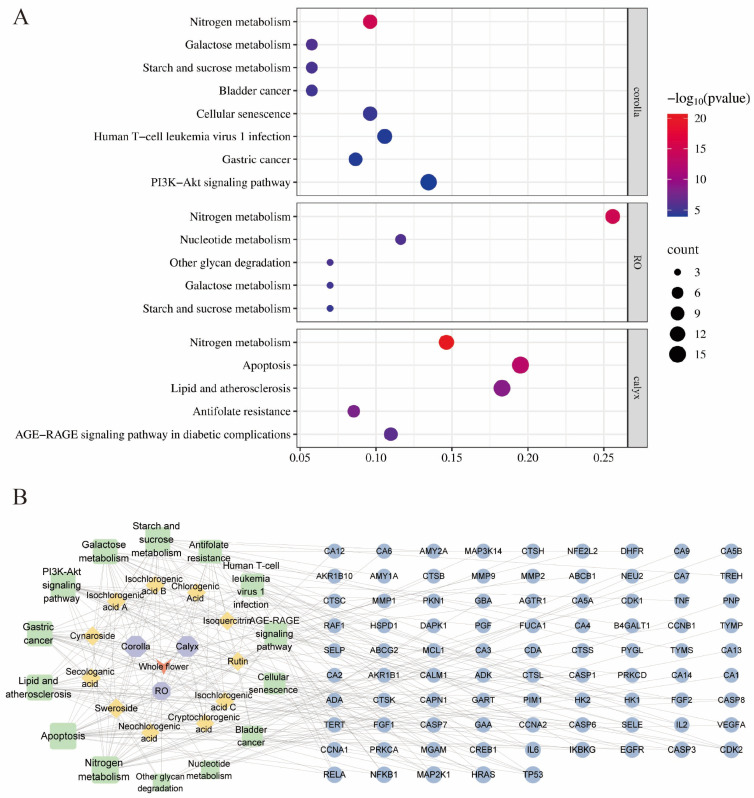
Prediction of targets based on the identified core functional components. (**A**) KEGG pathway enrichment analysis; (**B**) Construction of the component–target–pathway network. Node colors represent different categories: red, whole flower; light blue, floral organs; yellow, non-volatile compounds with significant correlations; green, involved signaling pathways; blue, functional targets.

To explore the potential molecular basis underlying the functional properties of different floral organs of LJF before and after flowering, a compound–target network was constructed based on previously identified differential metabolites. Protein–protein interaction (PPI) analysis and KEGG pathway enrichment analysis were subsequently performed to predict the potential biological pathways associated with each organ (Figure 9). The PPI network identified several core targets, including TNF, IL6, TP53, EGFR, CASP3, MMP9, PTGS2, and NFKB1. The compound–target network illustrated the associations between major constituents in different organs and their corresponding predicted targets. Flavonoids were mainly enriched in the corolla, whereas phenolic acids were predominant in the calyx, suggesting possible differences in functional contributions among floral organs.

KEGG pathway enrichment analysis revealed distinct pathway profiles among different organs. In the corolla, enriched pathways were mainly related to metabolic processes (e.g., nitrogen metabolism, galactose metabolism, and starch and sucrose metabolism), as well as pathways associated with cell growth and signal transduction, including the PI3K–Akt signaling pathway and cellular senescence. These results may suggest that certain constituents in the corolla are associated with processes related to metabolism and cell regulation, which could be relevant in the context of functional food development. In the calyx, enriched pathways were mainly involved in inflammation- and oxidative stress-related processes, including apoptosis, lipid and atherosclerosis, and the AGE–RAGE signaling pathway in diabetic complications. These pathways may be related to the traditionally described functions of heat-clearing and detoxifying, as well as anti-inflammatory and antioxidant effects. In the reproductive organs, enriched pathways were primarily associated with fundamental metabolic processes, such as nitrogen metabolism, nucleotide metabolism, glycan degradation, galactose metabolism, and starch and sucrose metabolism. These pathways are generally linked to energy supply and biosynthesis. Combined with the relatively higher levels of iridoids and their positive correlation with sugars observed in this study, this may suggest a potential association between active carbohydrate metabolism and secondary metabolite accumulation in these tissues.

Based on the differential metabolites, predicted targets, and enriched pathways of each floral organ, we constructed a compound–pathway–target network. Compounds derived from the corolla, including rutin, cynaroside, isoquercitrin, and swertiamarin, were mainly connected to targets such as TP53, EGFR, IL6, and TNF. These targets were further enriched in pathways including the PI3K-Akt signaling pathway, cellular senescence, bladder cancer, and gastric cancer. Calyx-derived compounds, primarily phenolic acids, were predominantly linked to inflammation-related targets such as NFKB1, CASP3, and MMP9, which were further enriched in pathways including apoptosis, lipid and atherosclerosis, as well as the AGE–RAGE signaling pathway in diabetic complications. This distribution is highly consistent with the traditional function of clearing heat and detoxifying. Compounds derived from the reproductive organs, particularly secologanic acid, were mainly associated with targets including GBA, MGAM, and ADA. The enriched pathways were primarily related to fundamental metabolic processes, such as nitrogen metabolism, galactose metabolism, and starch and sucrose metabolism. Additionally, the association between iridoids and carbohydrate metabolism is also suggested at the network level.

**Figure 10:**
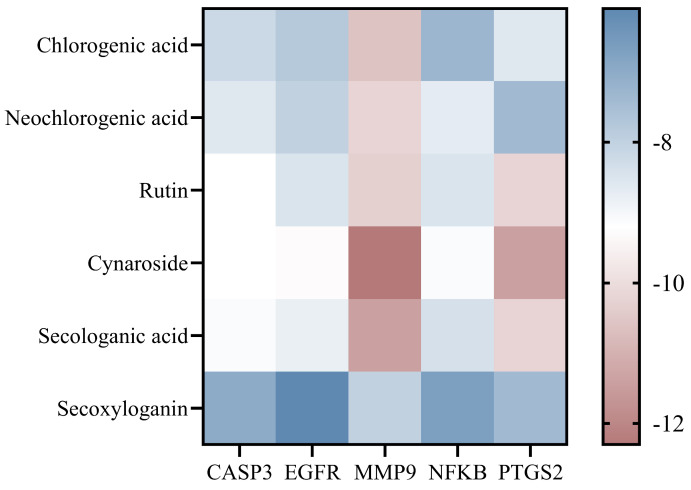
Heatmap of binding energies between small molecules and target proteins.

To validate the predictions derived from the network pharmacology analysis, six core compounds, including chlorogenic acid, neochlorogenic acid, rutin, cynaroside, secologanic acid, and secoxyloganin, were selected for molecular docking with five key targets, namely CASP3, EGFR, MMP9, NFKB1, and PTGS2. The results showed that all compounds exhibited strong binding affinities toward the selected targets, with binding energies ranging from −6.1 to −12.3 kcal/mol (Figure 10).

Cynaroside demonstrated the most prominent binding performance, with a binding energy of −12.3 kcal/mol for MMP9, while the binding energies with PTGS2, EGFR, CASP3, and NFKB1 were −11.4, −9.3, −9.2, and −9.1 kcal/mol, respectively, indicating a strong binding capacity. Rutin also showed favorable interactions, with binding energies of −10.3 and −10.2 kcal/mol toward MMP9 and PTGS2, respectively, and −9.2 kcal/mol toward CASP3. Secologanic acid exhibited binding energies of −11.4 and −10.2 kcal/mol with MMP9 and PTGS2, respectively, and −8.8 kcal/mol with EGFR. Chlorogenic acid and neochlorogenic acid also displayed generally strong binding affinities, with binding energies of −10.6 and −10.2 kcal/mol toward MMP9, respectively. Although secoxyloganin showed relatively lower binding energies ranging from −6.1 to −8.0 kcal/mol, it still maintained a moderate binding capacity.

**Figure 11:**
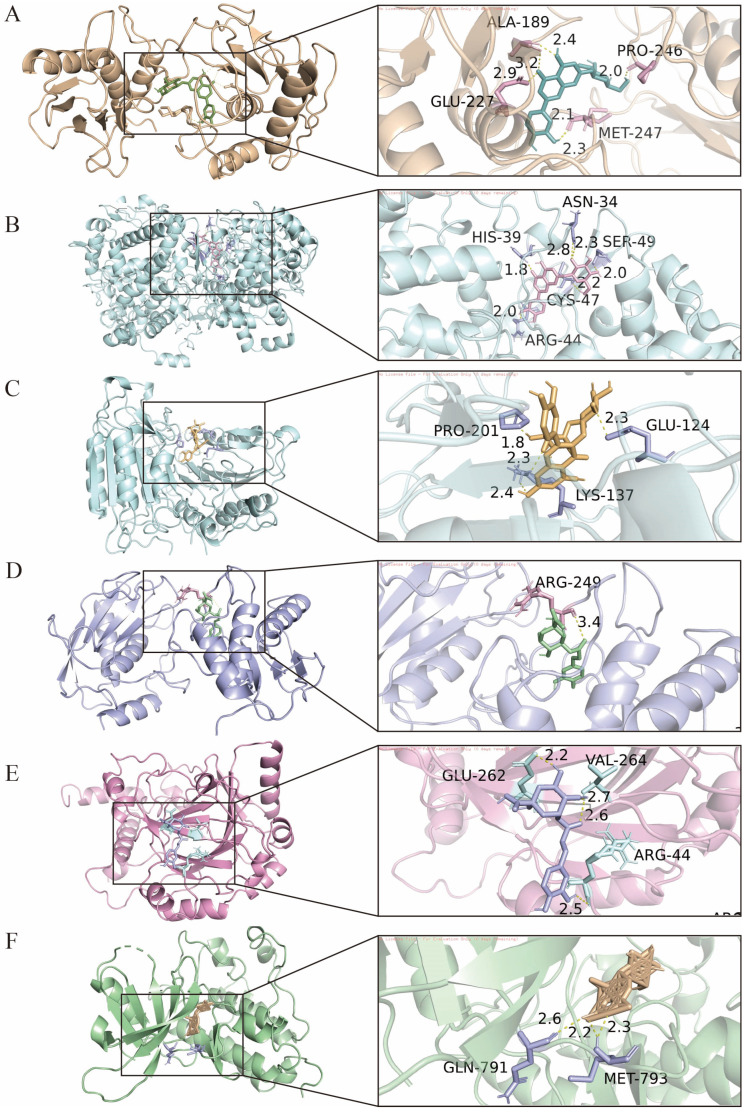
Molecular docking validation of key compounds with target proteins. (**A**) Binding mode of cynaroside with MMP9; (**B**) cynaroside with PTGS2; (**C**) rutin with CASP3; (**D**) chlorogenic acid with MMP9; (**E**) neochlorogenic acid with NFKB; (**F**) secologanic acid with EGFR.

The combinations demonstrating the strongest binding affinities were selected for binding mode analysis (Figure 11). Docking analysis of cynaroside with MMP9 indicated that the compound was situated within the active pocket, where its hydroxyl groups formed a hydrogen bond network with Met247, Pro246, Ala189, and Glu227. Given the enrichment of cynaroside in the corolla and the immunomodulation-related pathways predicted by network pharmacology in this study, it can be inferred that cynaroside may participate in the regulation of inflammatory responses and extracellular matrix homeostasis by inhibiting MMP9 activity, thus providing molecular evidence for the immunomodulatory function of *Lonicera japonica*. In the docking analysis between cynaroside and PTGS2, hydrogen bonds were formed with residues HIS39, ASN34, SER49, CYS47, and ARG44. Combined with the enrichment of cynaroside in the corolla and its strong binding affinity toward PTGS2 observed in this study, it is suggested that cynaroside may regulate inflammatory processes through inhibition of PTGS2 activity. Docking analysis of rutin with CASP3 showed interactions with active site residues including PRO201, GLU124, and LYS137.

Overall, the molecular docking results were highly consistent with the predictions from network pharmacology, structurally validating the binding capacity between the active components of LJF and the core targets. The strong binding of calyx-enriched phenolic acids, such as chlorogenic acid and neochlorogenic acid, to inflammation-related targets including NFKB1, PTGS2, and MMP9 supports the notion that the calyx is a principal contributor to the traditional detoxifying function. The strong multi-target interactions of corolla-enriched flavonoids, including cynaroside and rutin, with CASP3, EGFR, and MMP9 are consistent with the predicted immunomodulatory and cell signaling regulatory functions. Although iridoids in the reproductive organs are represented mainly by secologanic acid, their strong binding to multiple targets suggests potential synergistic multi-target regulatory effects in the metabolic processes of reproductive tissues.

## 4. Discussion

This study systematically elucidates the differences in flavor and functional metabolites among different floral organs of LJF before and after flowering, along with their implications for food applications. Both volatile and non-volatile components exhibited significant organ-specific distribution patterns. The calyx was enriched in phenolic acids and stable aldehydes, suggesting its potential contribution to overall flavor characteristics and antioxidant-related components, while the corolla preferentially accumulated flavonoids, which may be associated with functional properties. After flowering, the levels of phenolic acids and flavonoids decreased, whereas sugars and polysaccharides increased, indicating a shift in metabolic composition. Consequently, the bud stage may be more suitable as a source of antioxidant-related components, while fully opened flowers may show greater potential for development as sources of natural sweeteners, polysaccharide-enriched products, and foods targeting immune-related functions. By integrating entropy weight evaluation, correlation network analysis, and molecular docking results, this study provides a basis for the differentiated utilization of various floral organs of LJF, which may support its further development in plant-based functional foods and related products.

Developmental stage-dependent changes were observed, with phenolic acids and flavonoids enriched during the bud stage, while the content of sugars and polysaccharides increased after flowering. Similar trends have been reported in other plant systems. Coyago-Cruz et al. [31] found that during the ripening of *Solanum lycopersicum* L., sugar content gradually increased, whereas phenolic compounds exhibited a slight decreasing trend. Similarly, Jemai et al. [32] reported that key phenolic compounds, such as oleuropein, significantly decreased during olive fruit maturation, while sugar content increased in the later stages. These findings are consistent with the changes observed in LJF. Consequently, the substantial increase in polysaccharide content may be relevant to its potential functional utilization. Han et al. [15] reported that LJF polysaccharides (LP) exhibited immunomodulatory activity, including promoting spleen and thymus development in immunosuppressed mice and enhancing the secretion of cytokines (IL-2, IL-6, TNF-α) and immunoglobulins (IgA, IgG, IgM). The underlying mechanisms may be related to the regulation of gut microbiota and the production of short-chain fatty acids. These findings suggest that, compared with the conventional harvesting of flower buds for medicinal purposes, fully opened flowers may have greater potential for the development of functional foods with immunomodulatory potential.

The metabolic differences among floral organs further reveal the structural and functional specialization of LJF. Metabolites were not uniformly distributed; instead, they exhibited distinct organ-specific patterns. The relatively higher levels of phenolic acids and certain aldehydes in the calyx may be associated with its outer position and protective function. Liu et al. [33] reported that natural phenolic acids such as chlorogenic acid, gallic acid, and caffeic acid inhibit foodborne pathogens by disrupting cell membranes and inhibiting metabolic enzymes. Flavonoids were preferentially accumulated in the corolla, with rutin, cynaroside, and isoquercitrin exhibiting higher concentrations than in other organs (bud stage: 1.78–1.86, 0.46–0.59 mg/g DW, and 0.80–0.98 mg/g DW, respectively). In contrast, flavonoids were predominantly enriched in the corolla, which may be related to their potential roles in both sensory quality and bioavailability. Chen et al. [29] demonstrated that flavonoid aglycones (e.g., quercetin and isorhamnetin) derived from *Abelmoschus* corolla were rapidly absorbed following oral administration in mice, with exposure levels positively correlated with dosage and increased concentrations observed after repeated administration. These findings suggest that flavonoids enriched in the corolla may exhibit relatively favorable bioavailability. Meanwhile, the accumulation of sugars and certain primary metabolites was primarily observed in the corolla. This pattern may be associated with the role of the corolla in contributing to sensory quality and palatability. Sugars are key determinants of taste and can enhance the overall acceptability of edible floral tissues, which is particularly relevant in the context of functional food applications [34]. Additionally, previous studies have indicated that sugar accumulation during flowering is closely related to developmental regulation and carbohydrate allocation. Borghi et al. [35] reported that sugars and polysaccharides tend to increase after flowering, reflecting shifts in carbon partitioning during plant development. These results suggest that the enrichment of sugars in the corolla may be linked not only to developmental processes but also to its functional role as the primary edible and sensory-relevant tissue. Similar organ-specific metabolic distributions have been reported in other plant systems. Previous studies have shown that plant metabolites often display distinct spatial patterns closely associated with the physiological functions of specific tissues. For instance, Wen et al. [36] observed significant tissue-specific accumulation of multiple metabolites in different floral organs of *Areca catechu*, with certain bioactive compounds enriched in specific regions such as the ovary or ovules, reflecting their roles in development or protection. Taken together, these findings indicate that the metabolic differentiation among the floral organs of LJF represents a coordinated strategy of spatial metabolite allocation, contributing to the integration of structural functions and quality-related attributes. From a functional food perspective, such organ-specific compositional differences may provide a basis for the targeted utilization of different floral parts.

Volatile metabolites were also co-regulated by both developmental stage and organ differentiation. Prior to flowering, different floral parts had already established distinct aroma profiles, and these differences became further pronounced after flowering. Hierarchical clustering analysis (Figure 3) showed that the aroma profile of whole flowers after blooming increasingly resembled that of the reproductive organs, suggesting that the maturation of these organs plays an important role in shaping the overall flavor characteristics at the full-bloom stage. Similar patterns have been documented in other plant species. Yong et al. [37] found that the total content of volatile compounds in *Michelia crassipes* increased during flowering, with a higher abundance in pistils compared to other floral organs; the accumulation of benzenoid compounds in stamens may aid in pollinator attraction. These observations are generally consistent with the trends observed in LJF. 3-Decyn-2-ol was identified as a characteristic compound that accumulated specifically after flowering. It was nearly undetectable at the bud stage but exhibited an approximately 14-fold increase in reproductive organs after blooming. This compound has been reported to exhibit antimicrobial activity, and its preferential accumulation in reproductive tissues may suggest a potential role in protecting these organs from microbial stress or participating in post-flowering physiological processes [38]. Meanwhile, oleic acid was enriched in the corolla, showing an approximately 1.5-fold increase after flowering. As a major component of membrane phospholipids in eukaryotic cells, oleic acid may be associated with cell expansion and membrane biosynthesis during petal opening. It has also been recognized as a bioactive fatty acid in plants, suggesting its potential contribution to various physiological functions in floral tissues [39]. In contrast, basic aroma compounds such as 1-hexanol, 2-nonen-1-ol, and 1,7-octanediol were relatively uniformly distributed across different floral parts, forming the fundamental flavor profile of LJF. From a functional food perspective, these findings indicate that the flavor characteristics and potential functionalities of LJF can be influenced by selecting specific harvest stages or floral organs [40]. For instance, corolla tissues may be preferentially utilized for flavor-oriented applications, while fully opened flowers with enhanced aroma intensity may have potential for use in high-aroma food products.

After clarifying the distribution patterns of metabolites in LJF across developmental stages and different floral parts, this study further explored their potential functional associations through correlation analysis, network pharmacology, and molecular docking. The coordinated changes among primary metabolites (e.g., sugars), volatile compounds, and secondary metabolites suggest potential interactions among these metabolic pathways. At the bud stage, relatively low sugar levels accompanied by higher abundances of phenolic acids and aldehydes may indicate a metabolic tendency toward the accumulation of defense-related components [41]. The co-occurrence of non-volatile phenolic compounds and volatile aldehydes may contribute to a protective chemical profile. In contrast, after flowering, increased sugar accumulation and the formation of characteristic aroma compounds (e.g., 3-decyn-2-ol) reflect a metabolic shift that may enhance sensory attributes. Meanwhile, the marked increase in polysaccharides suggests additional value for functional food development, particularly in products targeting nutritional or health-promoting properties. Molecular docking analysis suggested that cynaroside exhibited strong binding affinities with inflammation-related targets, including MMP9 and PTGS2, with a binding energy of −12.3 kcal/mol. Liu et al. [42], in their study on the anti-atherosclerotic mechanism of *Ziziphora clinopodioides* Lam., also reported that flavonoid compounds such as cynaroside showed strong binding interactions with MMP9, suggesting that these compounds may exert anti-inflammatory and tissue-protective effects by modulating MMP9 activity. Similarly, Yang et al. [43] demonstrated that cynaroside could alleviate renal fibrosis by inhibiting inflammatory responses, with its mechanism linked to the regulation of MAPK-related signaling pathways. These findings provide supporting evidence that cynaroside may be involved in inflammation-related processes. From the perspective of functional food development, these results suggest a differentiated utilization strategy for LJF. Fully opened flowers, which have traditionally been underutilized, could be explored for the development of products enriched in sugars and polysaccharides, such as health-oriented beverages or naturally sweetened products, thereby improving resource utilization. In contrast, the calyx, characterized by relatively higher levels of phenolic acids and stable aldehyde compounds, may be suitable for applications such as natural preservatives or flavor-enhancing ingredients. By moving beyond a single medicinal-use paradigm, LJF may serve as a diversified functional raw material, which could not only improve the economic value of cultivation by utilizing flowering-stage resources but also support the broader application of traditional edible–medicinal plants in the functional food industry.

Although this study characterized the metabolic differences in LJF across developmental stages and floral organs using a multi-omics approach combined with molecular docking; however, several limitations should be acknowledged. The analysis primarily focused on metabolomic profiling and in silico target prediction, which means that the findings remain at a predictive level. Further validation using cell-based or in vivo models is necessary to confirm the biological relevance of the extracts and their key constituents. Additionally, samples were obtained from a single origin and cultivar. Since plant metabolism is influenced by geographical and environmental factors, future studies should include multiple regions and varieties to improve generalizability. Lastly, the implications for functional food development are based on compositional characteristics rather than actual product formulation or sensory evaluation. Integrating bioactivity validation, processing effects, and product-level assessment will be necessary for practical application.

## 5. Conclusions

This study systematically characterizes the differences in flavor and functional metabolites among different floral organs of LJF before and after flowering, along with their implications for food applications. Both volatile and non-volatile components demonstrated significant organ-specific distribution patterns. The calyx was found to be enriched in phenolic acids and stable aldehydes, suggesting its potential contribution to flavor characteristics and antioxidant-related components, while the corolla showed higher levels of flavonoids, which may be pertinent to its functional properties. After flowering, the levels of phenolic acids and flavonoids generally decreased, whereas those of sugars and polysaccharides increased, indicating a shift in metabolic composition. Consequently, the bud stage may be more suitable as a source of antioxidant-related components, while fully opened flowers may show greater potential for development as sources of natural sweeteners, polysaccharide-enriched products, and foods targeting immune-related functions. By integrating entropy weight evaluation, correlation network analysis, and molecular docking results, this study provides a quantitative foundation for the differentiated utilization of various floral organs of LJF. These findings may support its further development in the realm of plant-based functional foods and related products.

## Data Availability

The original contributions presented in this study are included in the article/Supplementary Materials. Further inquiries can be directed to the corresponding author.

## Supplementary Materials

The following supporting information can be downloaded at: https://www.mdpi.com/article/10.3390/foods15101619/s1, Table S1: Comprehensive Method Validation Results for 13 Bioactive Compounds.; Table S2: Mass spectrometry identification results of volatile constituents in LJF by GC-MS; Table S3: Summary of two-way ANOVA results for volatile compounds in LJF across developmental stages and floral organs; Table S4: Mass spectrometry identification results of the chemical constituents of LJF; Table S5: Summary of two-way ANOVA results for all variables non-volatile metabolites in LJF across developmental stages and floral organs; Table S6: Summary of two-way ANOVA results for sugar-related components in LJF across developmental stages and floral organs; Figure S1: MS/MS spectrum of 3,5-dicaffeoylquinic acid used for compound identification; Figure S2: MS/MS spectrum of luteolin 7-glucoside used for compound identification; Figure S3: MS/MS spectrum of secologanin used for compound identification.
