# Supplementary material for: Integrated Metabolomics and Molecular Docking Reveal Spatial and Developmental Variations in Flavor and Bioactive Constituents of Lonicera japonica Flos
Source: Foods. 2026 May 7;15(10):1619. doi: 10.3390/foods15101619 (PMC13206357; doi:10.3390/foods15101619)
Supplement: Supplementary file 1 [file foods-15-01619-s001.zip › foods-4257169-supplementary.pdf]

**Flavor and Bioactive Shifts in *Lonicera japonica* Flos Across Floral Organs and Developmental Stages: Implications for Functional Food Development**

Weiran Feng[a], Zongshuo Li[b], Xi Dai[a], Yanxia Shu[a], Chao Yu[a], Yanwen Wu[a], Weidong Li[a,*]

[a] *School of Chinese Materia Medica, Beijing University of Chinese Medicine, Beijing 102488, P. R. China*

[b] *School of Life Sciences, Beijing University of Chinese Medicine, Beijing 102488, China.*

[*]Corresponding author:

Weidong Li, School of Chinese Materia Medica, Beijing University of Chinese Medicine, Beijing 102488, P. R. China.

Email address: liweidong2005@126.com

Wenfu Ma, School of Life Sciences, Beijing University of Chinese Medicine, Beijing 102488, China.

Email address: wenfuma@bucm.edu.cn

## S2 Materials and methods (Supplementary Material)

### *S2.1 Method Validation for Quantitative Analysis*

#### *S2.1.1 Preparation of Standard Solutions*

Mixed standard solutions were prepared by accurately weighing appropriate amounts of reference compounds, including neochlorogenic acid, chlorogenic acid, cryptochlorogenic acid, isochlorogenic acids A, B, and C, rutin, luteoloside, isoquercitrin, loganic acid, sweroside, loganin, and secologanic acid. The standards were dissolved in 70% methanol to obtain stock solutions at known concentrations and further diluted to a series of appropriate concentrations for calibration. All solutions were filtered prior to HPLC analysis.

#### *S2.1.2 Calibration Curves and Linearity*

Calibration curves were constructed by plotting peak areas (Y) against corresponding concentrations (X) of each analyte. All compounds exhibited good linear relationships within the tested ranges, with correlation coefficients ($R^2$) greater than 0.998. Detailed regression equations, correlation coefficients, and linear ranges are summarized in Table S1.

#### *S2.1.3 Precision*

Instrument precision was evaluated by six consecutive injections of the same mixed standard solution. The relative standard deviations (RSDs) of peak areas for all analytes were below 1.0%, indicating good instrument precision.

#### *S2.1.4 Repeatability*

Repeatability was assessed by analyzing six independently prepared sample solutions. The RSD values of peak areas for all analytes were below 3.0%, demonstrating good method repeatability.

#### *S2.1.5 Stability*

The stability of sample solutions was evaluated at 0, 2, 4, 8, 12, and 24 h at room temperature.

The RSD values of peak areas for all analytes were below 2.0%, indicating that the sample solutions were stable within 24 h.

*S2.1.6 Recovery*

Recovery was evaluated by spiking known amounts of mixed standard solutions into pre-analyzed samples. The average recoveries of the analytes ranged from 96.2% to 104.6%, with RSD values below 6.0%, indicating good accuracy of the method.

*S2.1.7 Limits of Detection and Quantification*

The limits of detection (LOD) and quantification (LOQ) were estimated based on signal-to-noise ratios of 3 and 10, respectively.

*S2.2 Quantitative contribution analysis based on entropy weight method (EWM)*

The analysis was conducted following a structured workflow: (1) normalization of all indicators; (2) determination of indicator weights for each group using the entropy weight method (EWM); (3) calculation of comprehensive scores via a weighted linear summation approach.

*S2.2.1 Indicator normalization*

To eliminate dimensional differences among multiple indicators, the raw data, $x_{ij}$, were normalized before the analysis.

$$y_{ij} = \begin{cases} \frac{x_{ij}-\min(x_j)}{\max(x_j)-\min(x_j)}, & \text{Positive indicators} \\ \frac{\max(x_j)-x_{ij}}{\max(x_j)-\min(x_j)}, & \text{Negative indicators} \end{cases} \tag{S1}$$

where $i$=1, 2, …, m denotes the sample index, and $j$=1, 2, …, n denotes the indicator index.

*Calculation of component weights using the EWM*

The EWM was applied to calculate the objective weight of each component in the comprehensive evaluation based on information entropy.

The entropy value was calculated as follows:

$$E_j = -k\sum_{i=1}^{n} p_{ij}\ln(p_{ij}), k = \frac{1}{\ln n} \quad \text{(S2)}$$

$$p_{ij} = \frac{y_{ij}}{\sum_{i=1}^{n} y_{ij}} \quad \text{(S3)}$$

The weight of each component was calculated as follows:

$$w_j = \frac{1-E_j}{\sum_{j=1}^{m}(1-E_j)} \quad \text{(S4)}$$

where $i$ = 1, 2, …, m denotes the sample index, and $j$ = 1, 2, …, n denotes the indicator (or variable) index.

A higher weight indicates a greater contribution of the corresponding part to flavor characteristics and functional properties.

*S2.2.2 Comprehensive score calculation based on the standardized weighted linear summation method*

Based on the indicator weights obtained using EWM, standardized indicator values were multiplied by their corresponding weights and summed to obtain a comprehensive score.

$$S_i = \sum_{j=1}^{m} w_j \times y_{ij} \quad \text{(S5)}$$

where $S_i$ represents the comprehensive score of the $i$th sample, $w_j$ denotes the weight of the $j$th indicator, and $y_{ij}$ is the standardized value of the $j$th indicator for the $i$th sample.

A higher comprehensive score indicates a greater relative importance of the corresponding part.

**S3 Results and disscusion (Supplementary Material)**

*S3.1 MS/MS Fragmentation Analysis and Constituent Identification*

To ensure precise identification, the fragmentation pathways of major metabolites, including organic acids, flavonoids, and iridoids, were systematically investigated based on HRAM-MS/MS data.

*S3.1.1. Caffeoylquinic Acids (CQAs)*

CQAs are the predominant organic acids in LJF. In negative ion mode, these compounds typically formed prominent $[M\text{-}H]^-$ ions. The characteristic fragmentation involved the cleavage of the ester bond, resulting in the neutral loss of a caffeoyl moiety (162.0318 Da, $[C_9H_6O_3]$) and the formation of a quinic acid fragment at *m/z* 191.0560 ($[C_7H_{11}O_6]^-$).

Isomers such as 3-O-CQA (chlorogenic acid), 4-O-CQA (cryptochlorogenic acid), and 5-O-CQA (neochlorogenic acid) all exhibited the parent ion at *m/z* 353.0870. However, they were distinguished by their diagnostic fragments: 3-O-CQA and 5-O-CQA utilized *m/z* 191.0560 as the base peak, but 5-O-CQA uniquely displayed significant fragments at *m/z* 179.0349 ($[C_9H_7O_4]^-$) and 135.0451 ($[C_8H_7O_2]^-$). In contrast, 4-O-CQA was identified by its base peak at *m/z* 173.0454 ($[C_7H_9O_5]^-$, quinic acid minus $H_2O$).

Isomers including 3,5-di-O-CQA (isochlorogenic acid A), 3,4-di-O-CQA (isochlorogenic acid B), and 4,5-di-O-CQA (isochlorogenic acid C) showed parent ions at *m/z* 515.1189. The 4-substituted isomers (3,4-di-O-CQA and 4,5-di-O-CQA) followed a similar fragmentation to 4-O-CQA, yielding *m/z* 173.0454 as the base peak, whereas 3,5-di-O-CQA predominantly produced *m/z* 191.0560.

*S3.1.2. Flavonoids*

The flavonoids in LJF were primarily identified as O-glycosides. Their fragmentation initiated with the cleavage of the glycosidic bond to yield aglycone ions, followed by Retro-Diels-Alder (RDA) reactions of the C-ring to produce characteristic A-ring and B-ring fragments.

Compounds like luteoloside and lonicerin exhibited a neutral loss of hexose (162.0523 Da) or rutinoside (308.1102 Da), resulting in strong aglycone ions at *m/z* 285.0402 ($[C_{15}H_9O_6]^-$) and 284.0325 ($[C_{15}H_8O_6]^{-\bullet}$). Subsequent RDA fragmentation produced diagnostic ions at *m/z* 133.0294 ($[\text{A-ring}]^-$) and 151.0035 ($[\text{B-ring}]^-$).

Rutin and isoquercitrin followed a more complex C-ring cleavage after losing sugar moieties to form the *m/z* 301.0273 ($[C_{15}H_9O_7]^-$) ion. Key fragments included *m/z* 300.0273 (radical aglycone), *m/z* 271.0249 ($[\text{M-H-CO}]^-$), *m/z* 151.0035 (RDA fragment), and *m/z* 255.0300 (rearrangement fragment).

*S3.1.3. Iridoids*

Iridoids in LJF were mainly secologanin derivatives, forming $[\text{M-H}]^-$ or $[\text{M+HCOO}]^-$ ions. Fragmentation often involved the neutral loss of Glc (162.0523 Da), $H_2O$, $CO_2$, or $OCH_3$ groups.

Loganin and 7-epiloganin (*m/z* 389.1457) were distinguished by their aglycone fragments (*m/z* 227.0927). Loganin yielded diagnostic ions at *m/z* 127.0401 and 101.0243, while 7-epiloganin produced a characteristic fragment at *m/z* 179.0561.

Compounds such as secoxyloganic acid and secoxyloganin exhibited ring-opening structures, which primarily lost the glucose moiety followed by subsequent losses of $CO_2$ and $H_2O$.

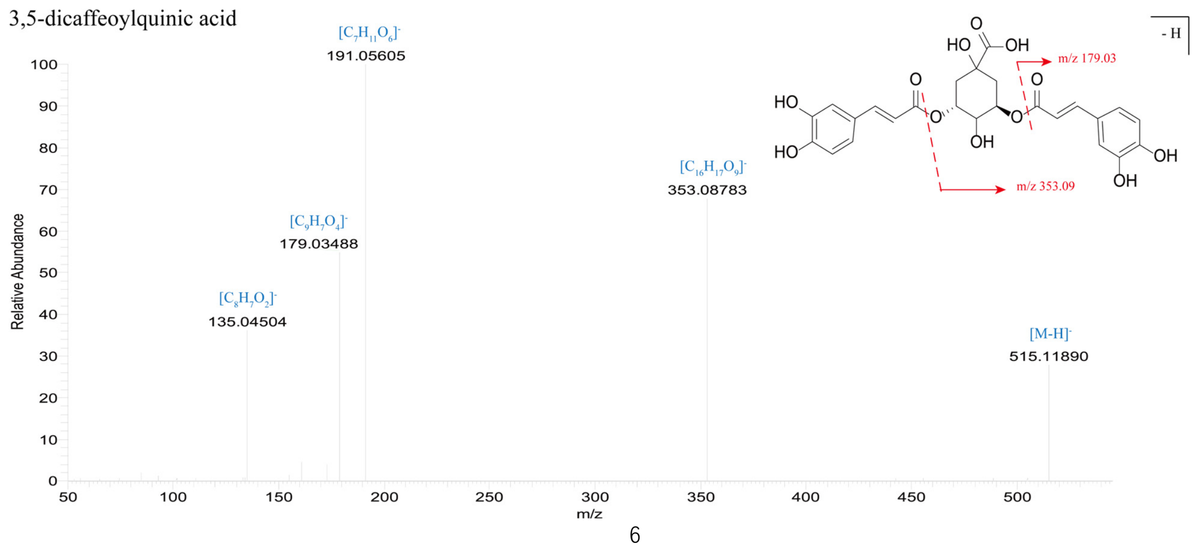

Figure S1 MS/MS spectrum of 3,5-dicaffeoylquinic acid used for compound identification.

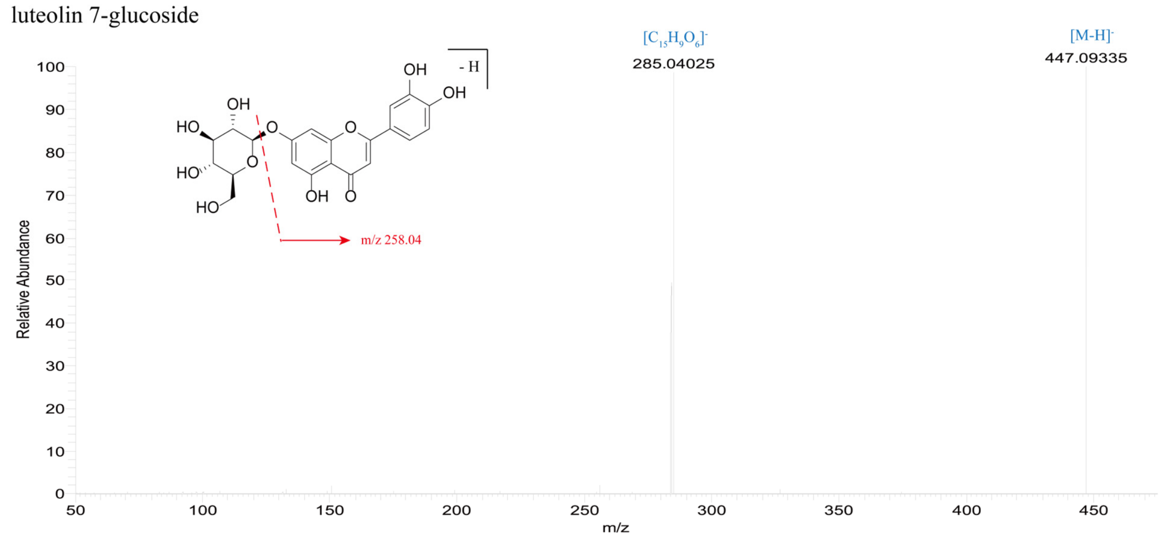


Figure S2 MS/MS spectrum of luteolin 7-glucoside used for compound identification.

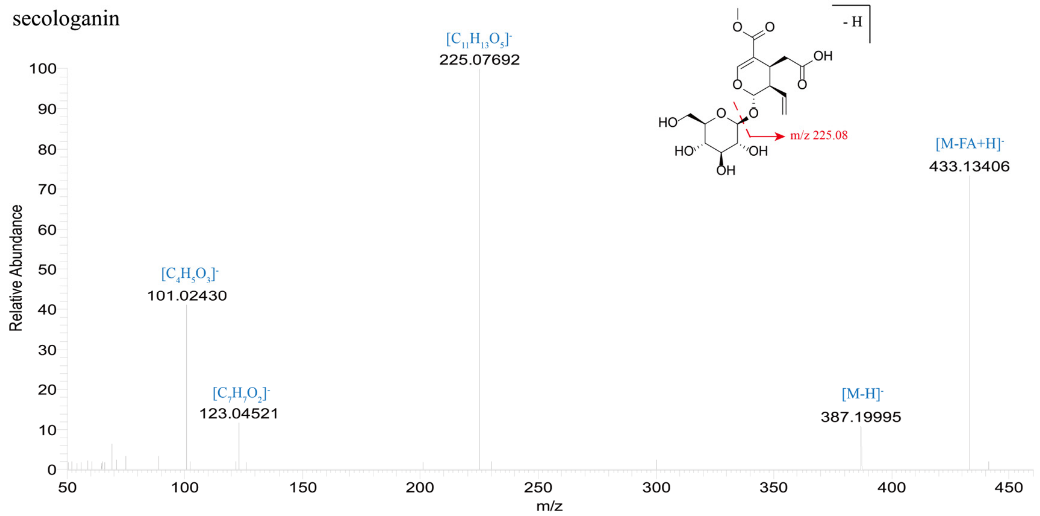


Figure S3 MS/MS spectrum of secologanin used for compound identification.

Table S1 Comprehensive Method Validation Results for 13 Bioactive Compounds.

| **Compound** | **Regression Equation** | $r^2$ | **Linear Range** /(μg·mL$^{-1}$) | **Precision (RSD %)** | **Repeatability (RSD %)** | **Recovery (%)** |
|---|---|---|---|---|---|---|
| Neochlorogenic acid | $Y = 29869705.54X - 8429.70$ | 0.9993 | 0.56–56.00 | 0.9 | 0.8 | 98.8 |
| Chlorogenic acid | $Y = 29390676.49X - 20859.83$ | 0.9999 | 30.00–3000.00 | 0.8 | 1.9 | 100.0 |
| Cryptochlorogenic acid | $Y = 22456585.40X - 11083.47$ | 0.9992 | 0.90–90.00 | 0.7 | 1.8 | 97.2 |
| Isochlorogenic acid B | $Y = 28425136.62X + 35669.67$ | 0.9991 | 23.20–2320 | 0.8 | 2.0 | 97.5 |
| Isochlorogenic acid A | $Y = 35368574.27X + 5223.43$ | 0.9999 | 2.80–280.00 | 0.8 | 1.8 | 101.8 |
| Isochlorogenic acid C | $Y = 35157929.95X + 17895.42$ | 0.9994 | 1.47–147.00 | 0.7 | 2.5 | 104.6 |
| Rutin | $Y = 13960191.90X - 7586.61$ | 0.9991 | 0.72–72.00 | 0.8 | 1.4 | 100.6 |
| Luteoloside | $Y = 28525360.49X - 11272.31$ | 0.9992 | 0.74–74.00 | 0.9 | 1.2 | 96.2 |
| Isoquercitrin | $Y = 18673173.01X - 11598.68$ | 0.9992 | 1.04–104.00 | 0.8 | 2.0 | 99.2 |
| Secoxyloganic acid | $Y = 11958161.81X + 325951.74$ | 0.9992 | 40.00–4000.00 | 0.7 | 1.6 | 98.7 |
| Sweroside | $Y = 16904962.82X - 11533.52$ | 0.9993 | 0.77–77.00 | 0.9 | 1.6 | 103.3 |
| Loganin | $Y = 14103047.04X - 7685.36$ | 0.9993 | 0.62–62.00 | 0.9 | 2.7 | 102.9 |

| | | | | | | |
|---|---|---|---|---|---|---|
| Loganic acid | $Y = 11343926.03X + 804777.17$ | 0.9987 | 6.14–614.00 | 1.0 | 1.8 | 100.5 |

Table S2 Mass spectrometry identification results of volatile constituents in LJF by GC-MS.

| No. | RT | Compound | pre-whole (%) | pre-corolla (%) | pre-foral organ (%) | pre-calyx (%) | post-whole (%) | post-corolla (%) | post-foral organ (%) | post-calyx (%) |
|---|---|---|---|---|---|---|---|---|---|---|
| 1 | 4.3 | isoamyl alcohol | 0.06±0.03 | 0±0 | 0.49±0.36333 | 0.26±0.15 | 0.21249±0.2273 | 0.03968±0.06873 | 0.07638±0.06199 | 0±0 |
| 2 | 6.1 | hexanal | 5.46±2.18 | 6.915±1.39 | 6.04667±0.6 | 7.38±0.28 | 7.21211±0.5153 | 5.69273±1.41979 | 6.37018±0.54993 | 7.93738±1.43855 |
| 3 | 6.5 | 2,3-butanediol | 1.78±1.24 | 1.12±0.8 | 3.63±1.46333 | 0.16±0.09 | 1.5453±0.8298 | 1.81993±0.99022 | 1.58418±1.3428 | 0.17286±0.1497 |
| 4 | 7.9 | 1-hexyn-3-ol | 0.34±0.09 | 0.98±0.36 | 0±0 | 0.3±0.28 | 0.35058±0.14052 | 0.07731±0.13391 | 0.11118±0.05606 | 0.58229±0.50428 |
| 5 | 8.4 | 3-methyl-4-pentenol | 0.22±0.09 | 0.19±0.16 | 0.43333±0.11667 | 0±0 | 0.27256±0.15499 | 0.21947±0.10399 | 0.52411±0.30712 | 0.26091±0.30293 |
| 6 | 8.9 | 1-Hexanol | 0.37±0.04 | 0.24±0.065 | 0.48333±0.06333 | 0.17±0.06 | 0.32664±0.09269 | 0.22781±0.06116 | 0.34646±0.06593 | 0.3033±0.00622 |
| 7 | 9.4 | 2-Heptanone | 0.24±0.05 | 0.25±0.045 | 0.21667±0.05667 | 0.17±0.03 | 0.32164±0.06907 | 0.24757±0.01121 | 0.27875±0.06071 | 0.25916±0.06429 |
| 8 | 9.8 | heptanal | 4.49±1.27 | 4.885±0.12 | 4.04667±0.64333 | 4.18±0.3 | 4.08626±0.47161 | 4.277±1.03914 | 4.07366±0.79255 | 4.27902±0.193 |

| | | | | | | | | | | |
|---|---|---|---|---|---|---|---|---|---|---|
| 9 | 11.9 | 1,7-octanediol | 0.2±0.07 | 0.26±0.055 | 0.16333±0.06 | 0.19±0.04 | 0.17948±0.08154 | 0.19357±0.01669 | 0.11414±0.01282 | 0.2689±0.02479 |
| 10 | 13.1 | trans-2-octen-1-Ol | 0.2±0.06 | 0.265±0.085 | 0.18667±0.07667 | 0.25±0.05 | 0.19622±0.02884 | 0.15521±0.04235 | 0.21583±0.02623 | 0.41726±0.10733 |
| 11 | 13.3 | 1-ethenoxy-6-methylheptane | 0.17±0.06 | 0.15±0.065 | 0.14333±0.04 | 0.21±0.08 | 0.15627±0.0059 | 0.16706±0.03932 | 0.14385±0.0291 | 0.11283±0.02906 |
| 12 | 13.5 | octanal | 4.74±1.03 | 5.13±0.535 | 4.46±0.69667 | 5.27±0.26 | 5.11964±0.32192 | 4.67479±0.51108 | 4.843±0.27805 | 5.22065±0.14519 |
| 13 | 15.5 | 1-octyn-3-ol | 0.53±0.01 | 0.56±0.045 | 0.45667±0.05333 | 0.6±0.03 | 0.5785±0.15187 | 0.53723±0.0786 | 0.51419±0.04084 | 0.54836±0.04057 |
| 14 | 16.9 | nonanal | 7.48±1.34 | 8.255±0.32 | 7.10333±0.73333 | 8.55±0.33 | 5.96607±5.07503 | 7.3943±0.87451 | 8.12156±0.1095 | 8.89154±0.46857 |
| 15 | 18.8 | trans-2-nonenal | 1.07±0.02 | 0.94±0.415 | 0.95±0.09333 | 1.12±0.02 | 1.05458±0.0745 | 0.90106±0.29731 | 1.00706±0.04473 | 1.26906±0.04651 |
| 16 | 20.2 | Decanal | 1.41±0.32 | 1.65±0.18 | 1.37333±0.19333 | 1.75±0.13 | 1.64673±0.13501 | 1.46065±0.21647 | 1.73241±0.24034 | 1.66592±0.15042 |
| 17 | 20.7 | Octanoic acid | 0.11±0.06 | 0.16±0.02 | 0.18667±0.12667 | 0.17±0.13 | 0.35243±0.05078 | 0.25982±0.08949 | 0.19491±0.1949 | 0.3156±0.27339 |
| 18 | 22.0 | 2-decenal, (E)- | 5.49±0.59 | 5.85±0.26 | 5.14667±0.33667 | 6.16±0.31 | 5.81113±0.20018 | 5.26826±0.61162 | 5.42907±0.13021 | 5.9923±0.00505 |
| 19 | 22.8 | 2-nonen-1-ol | 0.19±0.08 | 0.245±0.075 | 0.14±0.05667 | 0.28±0.04 | 0.22675±0.04326 | 0.11553±0.02452 | 0.17162±0.04518 | 0.1253±0.00747 |
| 20 | 23.4 | trans-2-undecen-1-o | 0.31±0.07 | 0.37±0.06 | 0.31±0.06667 | 0.52±0.07 | 0.42697±0.02844 | 0.36355±0.01099 | 0.3125±0.27291 | 0.51991±0.01677 |
| 21 | 25.3 | trans-2-undecenal | 5.85±0.57 | 6.335±0.175 | 5.56667±0.30667 | 6.62±0.26 | 4.27323±3.41879 | 5.62788±0.73424 | 5.9172±0.07864 | 6.47917±0.09722 |

| | | | | | | | | | | |
|---|---|---|---|---|---|---|---|---|---|---|
| 22 | 26.2 | 4-ethyl-1-octyn-3-ol | 0.19±0.06 | 0.22±0.015 | 0.20667±0.03 | 0.26±0.02 | 0.22159±0.02004 | 0.2018±0.02623 | 0.21836±0.07232 | 0.21906±0.03584 |
| 23 | 26.6 | dodecanal | 0.28±0.04 | 0.3±0.02 | 0.26333±0.04 | 0.36±0.08 | 0.3047±0.12632 | 0.25511±0.04618 | 0.26303±0.02895 | 0.31092±0.01797 |
| 24 | 28.4 | 2-dodecenal, (E)- | 0.76±0.07 | 0.815±0.065 | 0.63±0.14333 | 0.83±0.14 | 0.59543±0.10972 | 0.63605±0.02475 | 0.61991±0.04002 | 0.79851±0.00789 |
| 25 | 30.1 | 10-undecenal | 0.19±0.05 | 0.195±0.025 | 0.19±0.03333 | 0.25±0.1 | 0.25343±0.014 | 0.22198±0.0359 | 0.27335±0.02916 | 0.21303±0.00567 |
| 26 | 31.7 | cis-7-hexadecene | 0.52±0.1 | 0.41±0.045 | 0.35667±0.09667 | 0.57±0.17 | 0.39618±0.06022 | 0.36672±0.10431 | 0.44466±0.14288 | 0.52175±0.09979 |
| 27 | 32.9 | E-7-tetradecenol | 0.7±0.05 | 0.835±0.06 | 0.74±0.04667 | 0.47±0.15 | 0.80347±0.08402 | 0.49475±0.18598 | 0.94832±0.04939 | 0.25533±0.2877 |
| 28 | 33.6 | 2-(2-propynyloxy)cyclohexanol | 0.26±0.04 | 0.22±0.02 | 0.18667±0.08 | 0.28±0.04 | 0.19952±0.05695 | 0.18064±0.05063 | 0.19178±0.02116 | 0.22032±0.01388 |
| 29 | 34.2 | pentanoic acid, 10-undecenyl ester | 0.47±0.06 | 0.11±0.04 | 0.25±0.16 | 0.13±0.03 | 0.93843±0.72194 | 0.31142±0.33311 | 0.64318±0.64768 | 0.5345±0.71802 |
| 30 | 34.3 | cyclododecane | 1.12±0.22 | 1.115±0.415 | 1.19333±0.19333 | 1.4±0.02 | 1.07244±0.1784 | 1.23399±0.09734 | 1.13879±0.12271 | 1.24867±0.24062 |
| 31 | 35.0 | (Z)-9-tetradecenal | 0.85±0.1 | 1.005±0.035 | 0.86333±0.14 | 1±0.06 | 0.96699±0.11542 | 0.83393±0.12218 | 0.90305±0.05391 | 0.88165±0.07455 |
| 32 | 35.4 | palmitic acid | 0.46±0.04 | 0.495±0.035 | 0.44±0.07333 | 0.54±0.06 | 0.50249±0.04993 | 0.49231±0.05469 | 0.50283±0.01779 | 0.52969±0.00237 |
| 33 | 37.5 | cis-9-Hexadecenal | 0.83±0.08 | 0.93±0.07 | 0.74333±0.18333 | 1.03±0.06 | 0.92362±0.01227 | 0.88893±0.03795 | 0.69647±0.131 | 0.9002±0.01519 |

| | | | | | | | | | | |
|---|---|---|---|---|---|---|---|---|---|---|
| 34 | 40.9 | cyclopentaneundecanoic acid, methyl ester | 1.26±0.56 | 0.715±0.085 | 0.91667±0.10667 | 1.05±0.16 | 0.98456±0.063 | 1.24707±0.35429 | 1.04251±0.0935 | 1.2811±0.34107 |
| 35 | 41.9 | 17-octadecynoic acid | 0.15±0.11 | 0.195±0.13 | 0.15667±0.06 | 0.39±0.13 | 0.19091±0.1634 | 0.13615±0.0377 | 0.24445±0.15779 | 0±0 |
| 36 | 42.6 | oxacyclododecan-2-one | 0.13±0.02 | 0.245±0.095 | 0.25±0.15 | 0.35±0.15 | 0.11444±0.06525 | 0.18202±0.11344 | 0.1564±0.04474 | 0.17721±0.01425 |
| 37 | 44.6 | 13-octadecenal, (Z)- | 0.11±0.04 | 0.15±0.07 | 0.11±0.03667 | 0.1±0.03 | 0.07465±0.06576 | 0.08095±0.05147 | 0.09379±0.02249 | 0±0 |
| 38 | 44.9 | 9,12-octadecadienoic acid, methyl ester | 1.6±1.45 | 0.955±0.345 | 0.99333±0.25667 | 1.35±0.15 | 0.82177±0.36101 | 1.58964±0.53766 | 0.99102±0.04137 | 1.2524±0.02579 |
| 39 | 45.0 | 9,12,15-octadecatrienoic acid, methyl ester, (Z,Z,Z)- | 4.52±1.24 | 4.76±1.58 | 4.65±1.34667 | 5.77±0.17 | 4.11688±0.94933 | 4.75259±1.38282 | 4.74363±0.3712 | 5.54939±0.18747 |
| 40 | 45.6 | cis-9-octadecenoic acid, methyl este | 0.53±0.38 | 0.34±0.025 | 0.32±0.05667 | 0.37±0.03 | 0.34175±0.04679 | 0.38618±0.17406 | 0.30934±0.0194 | 0.32057±0.000608991 |
| 41 | 46.5 | oleic acid | 17.7±1.82 | 17.15±1.19 | 16.14667±1.14667 | 17.86±2.61 | 19.55341±0.76549 | 25.32503±1.4723 | 16.39528±1.0813 | 18.90442±0.99917 |

Table S3 Summary of two-way ANOVA results for volatile compounds in LJF across developmental stages and floral organs.

| Compound | Factor | F value | p value |
|---|---|---|---|

| | | | |
|---|---|---|---|
| 3-decyn-2-ol | Stage | 50.979 | < 0.001 |
| | Organ | 18.466 | < 0.001 |
| | Stage × Organ | 26.961 | < 0.001 |
| 1-Hexanol | Stage | 0.041 | 0.841 |
| | Organ | 14.998 | < 0.001 |
| | Stage × Organ | 5.608 | 0.008 |
| 1,7-octanediol | Stage | 0.412 | 0.530 |
| | Organ | 5.706 | 0.007 |
| | Stage × Organ | 2.532 | 0.094 |
| 2-nonen-1-ol | Stage | 9.532 | 0.007 |
| | Organ | 1.704 | 0.206 |
| | Stage × Organ | 8.925 | 0.001 |
| trans-2-undecen-1-o | Stage | 14.483 | 0.002 |
| | Organ | 15.219 | < 0.001 |
| | Stage × Organ | 5.274 | 0.013 |
| 2-dodecenal, (E)- | Stage | 8.841 | 0.009 |
| | Organ | 6.037 | 0.006 |

| | | | |
|---|---|---|---|
| | Stage × Organ | 1.577 | 0.234 |
| E-7-tetradecenol | Stage | 1.114 | 0.307 |
| | Organ | 13.375 | < 0.001 |
| | Stage × Organ | 5.120 | 0.011 |
| oleic acid | Stage | 22.831 | < 0.001 |
| | Organ | 7.653 | 0.002 |
| | Stage × Organ | 13.903 | < 0.001 |

Note: Two-way ANOVA was performed to evaluate the effects of developmental stage (Stage), floral organ (Organ), and their interaction (Stage × Organ).

Table S4 Mass spectrometry identification results of the chemical constituents of LJF.

| RT (min) | Compound | *m/z* | Adduct | MF | $MS^2$ information | upper | style | receptacle |
|---|---|---|---|---|---|---|---|---|
| 1.22 | sucrose | 341.10870 | [M–H]- | $C_6H_{22}O_{11}$ | 179.06,89.02,161.05,59.01 | √ | √ | √ |
| 1.23 | arginine | 175.11895 | [M+H]+ | $C_6H_{14}N_4O_2$ | 70.07,175.12,60.06,116.07,130.10,158.09 | √ | √ | √ |
| 1.25 | glutamine | 147.07618 | [M+H]+ | $C_5H_{10}N_2O_3$ | 84.04,130.05,101.07,147.08 | √ | √ | √ |
| 1.27 | gluconic acid | 195.04993 | [M–H]- | $C_6H_{12}O_7$ | 75.01,129.02,177.04,99.01,159.03,99.01 | √ | √ | √ |
| 1.28 | glutamic acid | 148.06043 | [M+H]+ | $C_5H_9NO_4$ | 84.04,102.5,130.5 | √ | √ | √ |
| 1.33 | proline | 116.07061 | [M+H]+ | $C_5H_9NO_2$ | 70.07 | √ | √ | √ |

| | | | | | | | | |
|---|---|---|---|---|---|---|---|---|
| 1.33 | malic acid | 133.01410 | [M–H]- | $C_4H_6O_5$ | 115.00,71.01,72.99,89.02 | √ | √ | √ |
| 1.36 | tyrosine | 182.08117 | [M+H]+ | $C_9H_{11}NO_2$ | 136.08,123.04,165.05,119.05,182.08 | √ | √ | √ |
| 1.37 | fumaric acid | 115.00360 | [M–H]- | $C_4H_4O_4$ | 71.01 | √ | √ | √ |
| 1.58 | uridine diphosphategalactose | 565.04803 | [M–H]- | $C_{15}H_{24}N_2O_{17}P_2$ | 78.96,323.03,96.97,158.92 | √ | √ | √ |
| 1.59 | methoxy-caffeoylquinic acid | 383.11877 | [M–H]- | $C_{17}H_{20}O_{10}$ | 191.06 | √ | √ | √ |
| 2.07 | valine | 118.08630 | [M+H]+ | $C_5H_{11}NO_2$ | 72.08,55.05 | √ | √ | √ |
| 2.09 | adenine | 136.06177 | [M+H]+ | $C_5H_5N_5$ | 136.06 | √ | √ | √ |
| 2.15 | citric acid | 191.01866 | [M–H]- | $C_6H_8O_7$ | 111.01,87.01,85.03,129.02,173.00 | √ | √ | √ |
| 2.45 | uridine | 243.06215 | [M–H]- | $C_9H_{12}N_2O_6$ | 110.02,200.6,152.04,82.03 | √ | √ | √ |
| 2.93 | erythrose-glucoside | 265.09256 | [M–H]- | $C_{10}H_{18}O_8$ | 89.02,59.01,119.03,71.01,101.02 | √ | √ | √ |
| 3.14 | isoleucine | 132.10191 | [M+H]+ | $C_6H_{13}NO_2$ | 86.10,72.94.69.07 | √ | √ | √ |
| 3.15 | adenosine | 312.09433 | [M+FA–H]- | $C_{10}H_{13}N_5O_4$ | 134.05,266.09 | √ | √ | √ |
| 3.20 | leucine | 132.10191 | [M+H]+ | $C_6H_{14}NO_2$ | 86.10,72.94,69.07 | √ | √ | √ |
| 3.74 | guanine | 152.05663 | [M+H]+ | $C_5H_5N_5O$ | 135.03,110.03 | √ | √ | √ |
| 3.88 | guanosine | 282.08417 | [M–H]- | $C_{10}H_{13}N_5O_5$ | 150.04,133.01 | √ | √ | √ |
| 3.98 | deoxyarabinitol-glucoside | 297.11877 | [M–H]- | $C_{11}H_{22}O_9$ | 179.06,161.04,135.06,119.03,101.02 | √ | √ | × |
| 7.00 | 3-hydroxybenzoic acid | 137.02428 | [M–H]- | $C_7H_6O_3$ | 93.03,65.04 | √ | √ | √ |
| 7.01 | phenylalanine | 166.08625 | [M+H]+ | $C_9H_{11}NO_2$ | 120.08,103.5 | √ | √ | √ |
| 7.31 | secologanoside or isomer | 389.10910 | [M–H]- | $C_{16}H_{22}O_{11}$ | 121.06,89.02,165.06,183.07,227.06 | √ | √ | √ |
| 7.39 | pantothenic acid | 218.10272 | [M–H]- | $C_9H_{17}NO_5$ | 146.08,88.04,71.01 | √ | √ | √ |
| 7.49 | gentisic acid 5-*O*-*β*-*D*-glucopyranoside | 315.07162 | [M–H]- | $C_{13}H_{16}O_9$ | 152.01,108.02,153.02,109.03 | √ | √ | √ |
| 7.57 | vanillic acid-glucoside | 329.08771 | [M–H]- | $C_{14}H_{18}O_9$ | 167.03,108.02,123.04,152.01,302.78 | √ | √ | √ |
| 7.60 | hydroxypropionyl-glc-fagomine or isomer | 380.15622 | [M–H]- | $C_{15}H_{27}NO_{10}$ | 146.08,308.14 | √ | √ | √ |
| 7.66 | swertiamarin | 419.11938 | [M+FA–H]- | $C_{16}H_{22}O_{10}$ | 211.06,408.31,147.12,181.05,328.26,300.24 | √ | √ | √ |
| 7.93 | caffeoylquinic acid-glc | 515.14022 | [M–H]- | $C_{22}H_{28}O_{14}$ | 191.06,179.03,161.02,135.04,323.08,353.09 | √ | √ | √ |
| 8.10 | syringic acid-glucoside | 359.09875 | [M–H]- | $C_{15}H_{20}O_{10}$ | 197.05,138.03,182.02,153.05,123.01 | √ | × | × |

| | | | | | | | | |
|---|---|---|---|---|---|---|---|---|
| 8.27 | loganic acid or isomer | 421.13525 | [M–H]- | $C_{16}H_{24}O_{10}$ | 89.02,59.01,241.07,375.07,389.11,179.06 | √ | √ | √ |
| 8.28 | 5'-methylthioadenosine | 298.09560 | [M+H]+ | $C_{11}H_{15}N_5O_3S$ | 136.06,145.03,163.04 | √ | √ | √ |
| 8.31 | 5-*O*-caffeoylquinic acid | 353.08765 | [M–H]- | $C_{16}H_{18}O_9$ | 191.06,179.03,135.04 | √ | √ | √ |
| 8.35 | hydroxybenzoic acid glucosider | 299.07693 | [M–H]- | $C_{13}H_{16}O_8$ | 137.02,93.03 | √ | √ | √ |
| 8.54 | feruloylquinic acid-glc | 529.15851 | [M–H]- | $C_{23}H_{30}O_{14}$ | 93.03,173.05,191.06,87.01,193.05,408.44 | √ | √ | √ |
| 8.71 | caffeoylquinic acid * | 353.08765 | [M–H]- | $C_{16}H_{18}O_9$ | 191.06,179.03,135.04 | × | × | √ |
| 8.82 | tryptophan | 205.09715 | [M+H]+ | $C_{11}H_{12}N_2O_2$ | 188.07,146.06,118.07,144.08 | √ | √ | √ |
| 8.87 | isomer of loganic acid | 375.12881 | [M–H]- | $C_{16}H_{24}O_{10}$ | 213.08,69.03,89.02,113.02,169.09,151.08 | √ | √ | √ |
| 8.92 | esculin | 339.07202 | [M–H]- | $C_{15}H_{16}O_9$ | 177.02 | √ | × | √ |
| 8.97 | tyrosine-dihydroferuloyl-glc | 538.19305 | [M–H]- | $C_{25}H_{33}NO_{12}$ | 180.07,408.42 | √ | × | × |
| 9.04 | caffeoylquinic acid-glc | 515.14020 | [M–H]- | $C_{22}H_{28}O_{14}$ | 191.06,323.08,161.02 | √ | √ | × |
| 9.38 | desacetylhookerioside | 581.16870 | [M+FA–H]- | $C_{22}H_{32}O_{15}$ | 535.17,179.06,373.11,89.02,303.07,445.13 | √ | × | √ |
| 9.39 | kingiside | 449.13013 | [M+FA–H]- | $C_{17}H_{24}O_{11}$ | 241.07,89.02,101.02,127.04,403.13,359.14 | √ | √ | √ |
| 9.48 | loganic acid * | 375.12933 | [M–H]- | $C_{16}H_{24}O_{10}$ | 69.03,59.01,89.02,151.08,119.03,195.07 | √ | √ | √ |
| 9.71 | morroniside | 451.14461 | [M+FA–H]- | $C_{17}H_{26}O_{11}$ | 243.09,405.14,101.02,179.06,141.06,155.03 | × | √ | √ |
| 9.94 | secologanoside or isomer | 389.10892 | [M–H]- | $C_{16}H_{22}O_{11}$ | 121.07,89.02,345.12,165.05,209.04,183.07 | √ | √ | √ |
| 10.05 | ferulic acid-glucoside | 355.10312 | [M–H]- | $C_{16}H_{20}O_9$ | 193.06 | × | × | √ |
| 10.07 | caffeic acid * | 178.03385 | [M–H]- | $C_9H_8O_4$ | 135.05 | √ | √ | √ |
| 10.07 | quinic acid | 191.05992 | [M–H]- | $C_7H_{12}O_6$ | 191.06 | √ | √ | √ |
| 10.07 | 3-*O*-caffeoylquinic acid | 353.08701 | [M–H]- | $C_{16}H_{18}O_9$ | 191.06 | √ | √ | √ |
| 10.07 | 5-deoxylamiol | 407.00711 | [M+FA–H]- | $C_{16}H_{26}O_9$ | 248.99,205.00,361.15,343.14,234.97,179.06 | √ | √ | × |
| 10.19 | 4-hydroxycinnamic acid glucoside | 325.09137 | [M–H]- | $C_{15}H_{18}O_8$ | 163.04,119.05 | √ | √ | √ |
| 10.23 | 6-hydroxyluteolin 7-*O*-diglucoside | 625.13995 | [M–H]- | $C_{27}H_{30}O_{17}$ | 299.02,301.03,462.08,271.03 | √ | √ | √ |
| 10.29 | secologanic acid-glc | 535.16656 | [M–H]- | $C_{22}H_{32}O_{15}$ | 97.03,113.02,89.02,193.05,373.11,408.41,515.19 | √ | √ | √ |

| | | | | | | | | |
|---|---|---|---|---|---|---|---|---|
| 10.36 | secologanoside A * | 507.17142 | [M–H]- | $C_{21}H_{32}O_{14}$ | 89.02,59.01,71.01,101.02,125.02,327.11,357.12 | √ | √ | √ |
| 10.43 | ferulic acid | 193.04991 | [M–H]- | $C_{10}H_{10}O_4$ | 178.03,134.06,149.06 | √ | √ | √ |
| 10.45 | secologanic acid * | 373.11365 | [M–H]- | $C_{16}H_{22}O_{10}$ | 89.02,193.05,97.03,59.01,69.03,149.06,71.01 | √ | √ | √ |
| 10.47 | 4-*O*-caffeoylquinic acid | 353.08792 | [M–H]- | $C_{16}H_{18}O_9$ | 191.06,173.04,179.03,135.04 | √ | √ | √ |
| 10.75 | secologanoside-7-methyl ester | 449.13010 | [M+FA–H]- | $C_{17}H_{24}O_{11}$ | 241.07,127.04,95.05,101.02,139.00,165.05 | √ | √ | √ |
| 10.84 | tetrahydro-methoxy-dimer caffeoylquinic acid | 739.20996 | [M–H]- | $C_{33}H_{40}O_{19}$ | 191.06,203.03,161.02 | √ | √ | √ |
| 10.85 | feruloyl-triglucoside | 743.21594 | [M+FA–H]- | $C_{28}H_{42}O_{20}$ | 341.11,697.22,89.02,119.03,179.06,588.00 | √ | √ | √ |
| 11.32 | tyrosine-loganin | 552.20782 | [M–H]- | $C_{26}H_{35}NO_{12}$ | 180.07,101.02,328.16,408.17,288.09,163.04 | √ | √ | √ |
| 11.84 | 1-*O*-caffeoylquinic acid | 353.08777 | [M–H]- | C16H18O9 | 191.06 | √ | √ | √ |
| 11.96 | caffeoylquinic acid-glc | 515.14020 | [M–H]- | $C_{22}H_{28}O_{14}$ | 167.07,101.02,,195.07,135.04,471.15 | √ | √ | × |
| 12.14 | vomifoliol-xyl-glc | 563.23425 | [M+FA–H]- | $C_{24}H_{38}O_{12}$ | 517.23,89.02,59.01,149.05,504.80,205.12 | √ | √ | √ |
| 12.18 | Loganin * | 435.14981 | [M+FA–H]- | $C_{17}H_{26}O_{10}$ | 227.09,101.02,127.04,389.15 | √ | √ | √ |
| 12.19 | Secologanin * | 433.13538 | [M+FA–H]- | $C_{17}H_{24}O_{10}$ | 225.08,101.02,123.04,387.13 | √ | √ | √ |
| 12.30 | Sweroside * | 403.12484 | [M–H]- | $C_{17}H_{24}O_{11}$ | 125.02,195.07,179.06,357.12 | √ | √ | √ |
| 12.32 | roseoside | 431.19254 | [M+FA–H]- | $C_{19}H_{30}O_8$ | 385.19,153.09,205.12,223.13,161.05 | √ | √ | √ |
| 12.37 | coumaryl-quinic acid | 337.09265 | [M–H]- | $C_{16}H_{18}O_8$ | 191.06,173.05,93.03,163.04,119.05,206.97 | √ | √ | √ |
| 12.80 | alpinoside | 415.12415 | [M–H]- | $C_{18}H_{24}O_{11}$ | 371.13,69.03,89.02,191.07,229.59,181.66 | √ | √ | √ |
| 13.04 | shimaurinoside B | 427.18155 | [M+FA–H]- | $C_{16}H_{30}O_{10}$ | 381.18,249.13,101.02,161.04,71.01 | √ | √ | √ |
| 13.15 | isomer of roseoside | 431.19229 | [M+FA–H]- | $C_{19}H_{30}O_8$ | 385.19,367.18,315.15,89.02,59.01,161.04 | √ | × | × |
| 13.16 | prolithospermoyl-quinic acid-quinic acid | 705.16724 | [M–H]- | $C_{32}H_{34}O_{18}$ | 513.10,339.05,191.06,295.06 | × | √ | √ |
| 13.29 | 7-epi-loganin | 435.15005 | [M+FA–H]- | $C_{17}H_{26}O_{10}$ | 179.06,101.02,89.02,227.09,157.05,389.15 | √ | √ | √ |
| 13.70 | indole-3-acetic acid-epiphlomurin | 594.21912 | [M–H]- | $C_{28}H_{37}NO_{13}$ | 276.09,244.06,286.11,216.07,201.06,507.56 | √ | √ | √ |
| 13.75 | 4-*O*-feruloylquinic acid | 367.10333 | [M–H]- | $C_{17}H_{20}O_9$ | 191.06,93.03,173.04,134.04 | √ | √ | √ |
| 13.91 | prolithospermoyl-quinic acid-caffeoylquinic acid or isomer | 867.19928 | [M–H]- | $C_{41}H_{40}O_{21}$ | 229.01,339.05,191.06,513.10 | × | √ | √ |

| | | | | | | | | |
|---|---|---|---|---|---|---|---|---|
| 13.97 | Secoxyloganin * | 403.12445 | [M–H]- | $C_{17}H_{24}O_{11}$ | 121.03,89.02,59.01,179.06,371.10,223.06 | √ | √ | √ |
| 14.40 | eucomoside C | 559.19391 | [M–H]- | $C_{27}H_{32}N_2O_{12}$ | 169.08,379.13,118.06,291.15,335.14 | √ | √ | √ |
| 14.79 | epivogeloside | 433.13571 | [M+FA–H]- | $C_{17}H_{24}O_{10}$ | 179.06,89.02,59.01,101.02,155.03,123.04 | √ | √ | √ |
| 14.82 | dihydro-secoxyloganin | 451.14658 | [M+FA–H]- | $C_{17}H_{26}O_{11}$ | 433.14,179.06,89.02,69.99,101.02,155.04 | √ | √ | √ |
| 15.06 | (7*R*,8*S*)-erythro-7,9,9'-trihydroxy-3,3'-dimethoxy-8-*O*-4'-neolignan-4-*O*-*β*-*D*-glucopyranoside | 585.74756 | [M+FA–H]- | $C_{26}H_{36}O_{12}$ | 179.06,89.02,59.01,119.03 | √ | √ | √ |
| 15.22 | 4'-*O*-*β*-*D*-glucopyranosyl-2'-hydroxyresveratrol 3-(7-*O*-*β*-*D*-glucopyranosyl-*β*-*D*-glucopyranoside) or isomer | 729.22528 | [M–H]- | $C_{32}H_{42}O_{19}$ | 89.02.101.02,229.08,281.12,453.14,504.83 | √ | √ | √ |
| 15.34 | caffeoylquinic acid methyl ester | 367.10345 | [M–H]- | $C_{17}H_{20}O_9$ | 191.06,135.04,,179.03,161.02 | √ | √ | √ |
| 15.44 | quercetin 3-*O*-arabinopyranosyl-(1'→6')-*β*-glucopyranoside | 595.13019 | [M–H]- | $C_{26}H_{28}O_{16}$ | 300.03,301.03,271.02,255.03,151.00 | √ | √ | √ |
| 15.46 | L-phenylalaninosecologanin | 536.21405 | [M–H]- | $C_{26}H_{35}NO_{11}$ | 164.07,147.05,69.03,101.02,228.10,272.09 | √ | √ | √ |
| 15.71 | 10-*O*-acetylgeniposide | 429.13992 | [M–H]- | $C_{19}H_{26}O_{11}$ | 153.05,69.03,101.02 | √ | √ | √ |
| 16.27 | lonicerin-xyl | 725.19336 | [M–H]- | $C_{32}H_{38}O_{19}$ | 285.04,284.03 | √ | √ | × |
| 16.32 | tryptophan-morroniside | 591.22180 | [M–H]- | $C_{28}H_{36}N_2O_{12}$ | 203.08,116.05 | √ | √ | √ |
| 16.67 | Rutin * | 609.14618 | [M–H]- | $C_{27}H_{30}O_{16}$ | 300.03,271.02,255.03,151.00 | √ | √ | √ |
| 16.75 | lonicerin-rha | 739.21051 | [M–H]- | $C_{33}H_{40}O_{19}$ | 285.04,284.03,191.06 | √ | √ | √ |
| 16.89 | dihydrodehydrodiconiferyl alcohol 4-*O*-*β*-*D*-glucopyranoside | 567.20898 | [M+FA–H]- | $C_{26}H_{34}O_{11}$ | 179.04,135.05,219.07,329.14,341.14,521.20 | √ | √ | √ |
| 16.97 | isomer of lonicerin | 593.15125 | [M–H]- | $C_{27}H_{30}O_{15}$ | 285.04,284.03,255.03,227.03 | √ | √ | √ |
| 17.06 | hyperoside | 463.08850 | [M–H]- | $C_{21}H_{20}O_{12}$ | 300.03,271.02,255.03,243.03,151.00 | × | √ | √ |
| 17.20 | luteolin-5-*O*-*β*-dglucopyranoside | 447.09321 | [M–H]- | $C_{21}H_{20}O_{11}$ | 285.04,284.03 | × | × | √ |
| 17.27 | threoninosecologanin | 456.15079 | [M–H]- | $C_{20}H_{27}NO_{11}$ | 224.06,276.09,294.10,122.02,180.07,122.2 | √ | √ | √ |
| 17.30 | Isoquercitrin * | 463.08829 | [M–H]- | $C_{21}H_{20}O_{12}$ | 300.03,271.03,255.03,151.00 | √ | √ | √ |

| | | | | | | | | |
|---|---|---|---|---|---|---|---|---|
| 17.43 | luteolin 7-*O*-*β*-*D*-xylosyl-(1→6)-*β*-*D*-glucopyranoside | 579.13556 | [M–H]- | $C_{26}H_{28}O_{15}$ | 285.04,284.03,255.03,227.03 | √ | × | √ |
| 17.49 | luteolin 7-glucoside | 447.09344 | [M–H]- | $C_{21}H_{20}O_{11}$ | 285.04,284.03 | √ | √ | √ |
| 17.56 | lonicerin | 593.15112 | [M–H]- | $C_{27}H_{30}O_{15}$ | 285.04,284.03 | √ | √ | √ |
| 17.59 | sylvestroside II | 821.27280 | [M+FA–H]- | $C_{34}H_{48}O_{20}$ | 89.02,775.27,743.24,155.03,101.02,119.03 | √ | √ | √ |
| 17.66 | eucomoside C | 559.19385 | [M–H]- | $C_{27}H_{32}N_2O_{11}$ | 169.08,291.15,335.14,273.14 | √ | √ | √ |
| 17.68 | 10-hydroxy-dec-2-enoic acid-glc-xyl | 479.21317 | [M–H]- | $C_{21}H_{36}O_{12}$ | 101.02,71.01,131.03,303.18,347.17,161.04 | √ | √ | √ |
| 17.71 | 10-*O*-acetylgeniposide | 429.13992 | [M–H]- | $C_{19}H_{26}O_{11}$ | 197.04,153.05,121.03,101.02,249.08 | √ | √ | √ |
| 17.82 | 5*α*-carboxystrictosidine | 573.20805 | [M–H]- | $C_{28}H_{34}N_2O_{11}$ | 393.15,89.02,142.07,185.11,317.13,361.12 | √ | √ | √ |
| 17.88 | secologanoyl loganic acid | 731.24005 | [M–H]- | $C_{32}H_{44}O_{19}$ | 375.13,89.02,195.07,300.11,650.34 | √ | √ | √ |
| 18.07 | hexyl-xyl-glc | 441.19830 | [M+FA–H]- | $C_{17}H_{32}O_{10}$ | 395.19,263.15,101.02,71.01,161.05,113.02 | √ | √ | √ |
| 18.37 | quercetin 3-*O*-*β*-*D*-(6''-*O*-malonyl)-glucopyranoside | 549.17285 | [M–H]- | $C_{24}H_{22}O_{15}$ | 505.10,300.03,301.03,271.02 | √ | × | √ |
| 18.42 | 3,4-dicaffeoylquinic acid * | 515.11798 | [M–H]- | $C_{25}H_{24}O_{12}$ | 191.06,179.04,173.05,135.05,353.09,135.04 | √ | √ | √ |
| 18.43 | kaempferol-3-*O*-rutinoside | 593.15112 | [M–H]- | $C_{27}H_{30}O_{15}$ | 285.04,284.03,255.03,227.04 | √ | √ | √ |
| 18.74 | tamaraxetin 3-*O*-neohesperidoside | 623.16113 | [M–H]- | $C_{28}H_{32}O_{16}$ | 299.02,314.05,315.05,271.03,498.60 | √ | √ | × |
| 18.74 | secologanoyl loganic acid or isomer | 731.24023 | [M–H]- | $C_{32}H_{44}O_{19}$ | 375.13,89.02,101.02,195.07,151.08,588.95 | √ | √ | √ |
| 18.84 | madreselvin A | 623.16168 | [M–H]- | $C_{28}H_{32}O_{16}$ | 315.05,300.03,299.02,271.02,243.03 | √ | √ | √ |
| 18.89 | 3,5-dicaffeoylquinic acid * | 515.11945 | [M–H]- | $C_{25}H_{24}O_{12}$ | 191.06,353.09,179.03,135.05 | √ | √ | √ |
| 18.93 | prolithospermoyl-quinic acid-caffeoylquinic acid | 867.19873 | [M–H]- | $C_{41}H_{40}O_{21}$ | 229.01,339.05,191.06,321.04,295.06,736.50 | √ | √ | √ |
| 19.03 | astragalin | 447.09344 | [M–H]- | $C_{21}H_{20}O_{11}$ | 285.04,284.03,255.03,227.03 | √ | √ | √ |
| 19.29 | 6-hydroxychrysoeriol 7-*O*-glucoside or isomer | 477.10391 | [M–H]- | $C_{22}H_{22}O_{12}$ | 314.04,271.02,243.03,285.04,257.05,299.02 | √ | √ | √ |
| 19.38 | 3-oxo-ionol glucoside | 415.19696 | [M+FA–H]- | $C_{19}H_{30}O_7$ | 179.06,89.02,119.03,369.19 | √ | √ | √ |
| 19.45 | apigenin 7-*O*-glucoside | 431.09866 | [M–H]- | $C_{21}H_{20}O_{10}$ | 268.04,269.04 | √ | √ | √ |
| 19.47 | eugenyl *β*-primeveroside | 503.17578 | [M+FA–H]- | $C_{21}H_{30}O_{11}$ | 293.09,89.02,71.01,457.17,125.02,148.05, | √ | √ | √ |

| | | | | | | | | |
|---|---|---|---|---|---|---|---|---|
| 19.84 | 4,5-dicaffeoylquinic acid | 515.11908 | [M–H]- | $C_{25}H_{24}O_{12}$ | 173.05,179.04,353.09,191.06,135.05 | √ | √ | √ |
| 19.95 | dihydro-methoxy-caffeoyl-dicaffeoylquinic acid | 709.19958 | [M–H]- | $C_{35}H_{34}O_{16}$ | 547.17,191.06,173.05,670.42 | √ | √ | √ |
| 20.03 | chrysoeriol-glc | 461.10980 | [M–H]- | $C_{22}H_{22}O_{11}$ | 255.03,283.02,298.05,446.09 | √ | √ | √ |
| 20.08 | 5*α*-carboxystrictosidine | 573.20880 | [M–H]- | $C_{28}H_{34}N_2O_{11}$ | 169.08,317.13,122.02,349.16,393.15,265.10 | √ | √ | √ |
| 20.17 | centauroside | 757.25598 | [M–H]- | $C_{34}H_{46}O_{19}$ | 89.02,101.02,595.20,179.06,119.03,525.16 | √ | √ | √ |
| 20.33 | secoxyloganin-diglucoside | 729.22424 | [M–H]- | $C_{29}H_{46}O_{21}$ | 97.03,373,11,193.05,149.06 | √ | √ | × |
| 20.50 | coumaryl-caffeoylquinic acid | 499.12402 | [M–H]- | $C_{25}H_{24}O_{11}$ | 163.04,191.06,353.09,337.09,119.05,135.04 | √ | √ | √ |
| 20.56 | prolithospermoyl-quinic acid-caffeoylquinic acid | 867.19763 | [M–H]- | $C_{41}H_{40}O_{21}$ | 339.05,513.10,191.06,295.06 | √ | √ | √ |
| 20.59 | hydroxynonanoic acid-quinic acid-caffeoylquinic acid | 683.25647 | [M–H]- | $C_{32}H_{44}O_{16}$ | 173.05,347.17,521.22 | √ | √ | √ |
| 20.59 | isoquercitrin-10-*O*-acetylgeniposide | 875.22435 | [M–H]- | $C_{41}H_{40}O_{21}$ | 300.03,301.03,463.09,271.02,151.00,588.61 | √ | √ | √ |
| 20.62 | dihydro-hydroxy-glc-glc-caffeoylquinic acid | 695.16052 | [M–H]- | $C_{28}H_{40}O_{20}$ | 191.06,179.03,135.05,353.09 | √ | √ | √ |
| 20.68 | lonijapospiroside B | 560.17694 | [M–H]- | $C_{27}H_{31}NO_{12}$ | 252.07,208.08,69.00,101.02,284.09,328.08 | √ | √ | √ |
| 20.70 | prolithospermoyl-quinic acid-dicaffeoylquinic acid | 1029.23035 | [M–H]- | $C_{50}H_{46}O_{24}$ | 339.05,513.10,191.06,295.06,495.09,675.13 | √ | √ | √ |
| 20.91 | pectolinarigenin 7-*O*-hexosylglucoside | 637.17700 | [M–H]- | $C_{29}H_{34}O_{16}$ | 329.07,328.06,313.03,314.05,299.02 | √ | √ | √ |
| 20.92 | tricaffeoylquinic acid | 677.15112 | [M–H]- | $C_{34}H_{30}O_{15}$ | 173.04,179.03,191.06,353.09,135.04,161.02 | √ | √ | √ |
| 21.05 | abscisic acid | 263.12930 | [M–H]- | $C_{15}H_{20}O_4$ | 219.14,157.08,204.12,139.08 | √ | √ | √ |
| 21.10 | Luteolin * | 285.04007 | [M–H]- | $C_{15}H_{10}O_6$ | 133.03,151.00.175.04,217.05 | √ | √ | √ |
| 21.15 | Quercetin * | 301.03476 | [M–H]- | $C_{15}H_{10}O_7$ | 151.00,179.00,107.01,121.03 | √ | √ | √ |
| 21.45 | tetrahydrodeoxycordifoline lactam | 541.18237 | [M–H]- | $C_{27}H_{30}N_2O_{10}$ | 122.02,265.10,361.12,309.09 | √ | √ | √ |
| 21.82 | apigenin | 269.04492 | [M–H]- | $C_{15}H_{10}O_5$ | 117.03,151.00,149.02,228.99,249.00,225.06 | √ | √ | √ |
| 22.03 | tricin | 329.06638 | [M–H]- | $C_{17}H_{14}O_7$ | 299.02,314.04,271.024 | √ | √ | √ |
| 22.09 | chrysoeriol | 299.05501 | [M–H]- | $C_{16}H_{12}O_4$ | 284.03,256.04 | √ | √ | √ |

| | | | | | | | | |
|---|---|---|---|---|---|---|---|---|
| 25.01 | prunetin | 283.06097 | [M–H]- | $C_{16}H_{12}O_3$ | 268.04 | √ | √ | √ |
| 29.03 | linolenic acid | 279.23169 | [M+H]+ | $C_{18}H_{30}O_2$ | 81.07,67.05,95.09,109.10,261.22 | √ | √ | √ |
| 29.38 | linoleic acid | 281.24751 | [M+H]+ | $C_{18}H_{32}O_2$ | 83.09,69.07,97.10,95.09,263.24,245.23 | √ | × | √ |
| 30.14 | stearic acid | 283.26428 | [M–H]- | $C_{18}H_{35}O_2$ | 283.26 | √ | × | √ |
| 30.95 | palmitic acid | 255.23282 | [M–H]- | $C_{16}H_{31}O_2$ | 255.23 | √ | × | √ |
| 32.30 | ursolic acid or oleanolic acid | 455.35197 | [M–H]- | $C_{30}H_{48}O_3$ | 456.35,356.16 | √ | √ | √ |

Note: Compounds marked with "*" were confirmed by comparison with authentic reference standards (including retention time and MS/MS spectra). Other compounds were putatively identified based on accurate mass measurements (mass error < 5 ppm), MS/MS fragmentation patterns, database matching (METLIN, MassBank, HMDB), and comparison with literature data.

Table S5 Summary of two-way ANOVA results for all variables non-volatile metabolites in LJF across developmental stages and floral organs.

| **Compound** | **Factor** | **F value** | **p value** |
|---|---|---|---|
| Neochlorogenic acid | Stage | 17.535 | < 0.001 |
| | Organ | 97.982 | < 0.001 |
| | Stage × Organ | 4.033 | 0.026 |
| Chlorogenic acid | Stage | 24.988 | < 0.001 |
| | Organ | 214.851 | < 0.001 |
| | Stage × Organ | 3.823 | 0.032 |
| Cryptochlorogenic acid | Stage | 1.587 | 0.226 |
| | Organ | 44.970 | < 0.001 |
| | Stage × Organ | 1.893 | 0.171 |
| Isochlorogenic acid B | Stage | 0.561 | 0.466 |
| | Organ | 74.697 | < 0.001 |
| | Stage × Organ | 2.384 | 0.110 |
| Isochlorogenic acid A | Stage | 12.745 | 0.003 |
| | Organ | 188.986 | < 0.001 |

| | | | |
|---|---|---|---|
| | Stage × Organ | 0.698 | 0.567 |
| Isochlorogenic acid C | Stage | 1.814 | 0.197 |
| | Organ | 154.707 | < 0.001 |
| | Stage × Organ | 1.099 | 0.378 |
| Total Phenolic acids | Stage | 64.972 | < 0.001 |
| | Organ | 521.761 | < 0.001 |
| | Stage × Organ | 3.674 | 0.036 |
| Rutin | Stage | 10.357 | 0.005 |
| | Organ | 57.306 | < 0.001 |
| | Stage × Organ | 3.165 | 0.053 |
| Cynaroside | Stage | 51.444 | < 0.001 |
| | Organ | 83.241 | < 0.001 |
| | Stage × Organ | 7.986 | 0.002 |
| Isoquercitrin | Stage | 68.569 | < 0.001 |
| | Organ | 170.191 | < 0.001 |
| | Stage × Organ | 18.499 | < 0.001 |
| Total Flavonoids | Stage | 35.054 | < 0.001 |

| | | | |
|---|---|---|---|
| | Organ | 102.814 | < 0.001 |
| | Stage × Organ | 5.172 | 0.014 |
| Secologanic acid | Stage | 14.300 | 0.002 |
| | Organ | 13.056 | < 0.001 |
| | Stage × Organ | 18.314 | < 0.001 |
| Sweroside | Stage | 2.401 | 0.141 |
| | Organ | 27.052 | < 0.001 |
| | Stage × Organ | 1.439 | 0.269 |
| Loganin | Stage | 5.527 | 0.032 |
| | Organ | 12.869 | < 0.001 |
| | Stage × Organ | 15.753 | < 0.001 |
| Secoxyloganin | Stage | 42.323 | < 0.001 |
| | Organ | 11.334 | < 0.001 |
| | Stage × Organ | 10.828 | < 0.001 |
| Total Iridoids | Stage | 2.940 | 0.106 |
| | Organ | 31.322 | < 0.001 |
| | Stage × Organ | 6.437 | 0.005 |

Note: Two-way ANOVA was performed to evaluate the effects of developmental stage (Stage), floral organ (Organ), and their interaction (Stage × Organ).

Table S6 Summary of two-way ANOVA results for sugar-related components in LJF across developmental stages and floral organs.

| **Compound** | **Factor** | **F value** | **p value** |
|---|---|---|---|
| Total sugar | Stage | 96.032 | < 0.001 |
| | Organ | 27.903 | < 0.001 |
| | Stage × Organ | 17.392 | < 0.001 |
| Reducing sugar | Stage | 195.375 | < 0.001 |
| | Organ | 93.922 | < 0.001 |
| | Stage × Organ | 27.192 | < 0.001 |
| Polysaccharide | Stage | 28.031 | < 0.001 |
| | Organ | 7.042 | 0.003 |
| Loganic acid | Stage × Organ | 10.112 | < 0.001 |

Note: Two-way ANOVA was performed to evaluate the effects of developmental stage (Stage), floral organ (Organ), and their interaction (Stage × Organ).
